# Impact of sphingosine and acetylsphingosines on the aggregation and toxicity of metal-free and metal-treated amyloid-β†

**DOI:** 10.1039/d0sc04366d

**Published:** 2020-12-17

**Authors:** Yelim Yi, Yuxi Lin, Jiyeon Han, Hyuck Jin Lee, Nahye Park, Geewoo Nam, Young S. Park, Young-Ho Lee, Mi Hee Lim

**Affiliations:** Department of Chemistry, Korea Advanced Institute of Science and Technology (KAIST) Daejeon 34141 Republic of Korea miheelim@kaist.ac.kr; Research Center of Bioconvergence Analysis, Korea Basic Science Institute (KBSI) Ochang Chungbuk 28119 Republic of Korea mr0505@kbsi.re.kr; Department of Chemistry Education, Kongju National University Gongju 32588 Republic of Korea; Department of Chemistry, Ulsan National Institute of Science and Technology (UNIST) Ulsan 44919 Republic of Korea; Research Headquarters, Korea Brain Research Institute (KBRI) Daegu 41068 Republic of Korea; Bio-Analytical Science, University of Science and Technology (UST) Daejeon 34113 Republic of Korea; Graduate School of Analytical Science and Technology, Chungnam National University Daejeon 34134 Republic of Korea

## Abstract

Pathophysiological shifts in the cerebral levels of sphingolipids in Alzheimer's disease (AD) patients suggest a link between sphingolipid metabolism and the disease pathology. Sphingosine (SP), a structural backbone of sphingolipids, is an amphiphilic molecule that is able to undergo aggregation into micelles and micellar aggregates. Considering its structural properties and cellular localization, we hypothesized that SP potentially interacts with amyloid-β (Aβ) and metal ions that are found as pathological components in AD-affected brains, with manifesting its reactivity towards metal-free Aβ and metal-bound Aβ (metal–Aβ). Herein, we report, *for the first time*, that SP is capable of interacting with both Aβ and metal ions and consequently affects the aggregation of metal-free Aβ and metal–Aβ. Moreover, incubation of SP with Aβ in the absence and presence of metal ions results in the aggravation of toxicity induced by metal-free Aβ and metal–Aβ in living cells. As the simplest acyl derivatives of SP, *N*-acetylsphingosine and 3-*O*-acetylsphingosine also influence metal-free Aβ and metal–Aβ aggregation to different degrees, compared to SP. Such slight structural modifications of SP neutralize its ability to exacerbate the cytotoxicity triggered by metal-free Aβ and metal–Aβ. Notably, the reactivity of SP and the acetylsphingosines towards metal-free Aβ and metal–Aβ is determined to be dependent on their formation of micelles and micellar aggregates. Our overall studies demonstrate that SP and its derivatives could directly interact with pathological factors in AD and modify their pathogenic properties at concentrations below and above critical aggregation concentrations.

## Abstract 2

The reactivity of sphingosine and acetylsphingosines towards both metal-free and metal-treated amyloid-β is demonstrated showing a correlation of their micellization properties.

## Graphical abstract

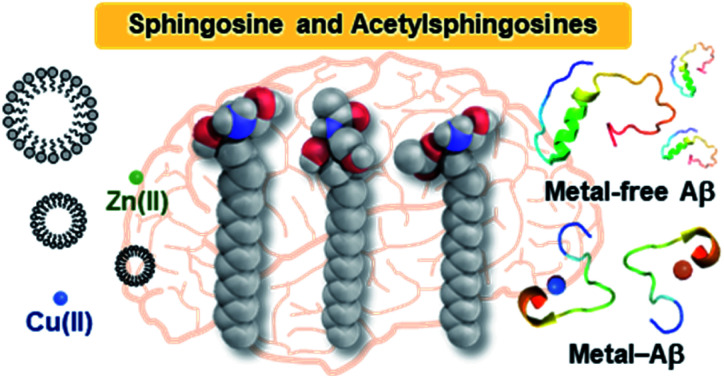

## Introduction

Accounting for approximately 50% of the brain's dry weight, lipids serve as signaling molecules, energy storage, and the building blocks of cellular membranes in neurons.^1,2^ Sphingolipids, among the different types of lipids, are essential for the structural integrity of neuronal membranes with implications in cellular recognition, neurotransmission, myelin sheath formation, and apoptotic regulation.^3,4^ Previous research has examined the pertinence of sphingolipid metabolism in brain homeostasis through the concept of sphingolipid rheostat, defined as the balance in the salvage pathway of sphingolipids.^5,6^ For these reasons, multiple aspects of sphingolipids have been investigated with respect to the development, function, and homeostasis of the brain as well as neurodegeneration.^3,5,7–10^

**Fig. 1:**
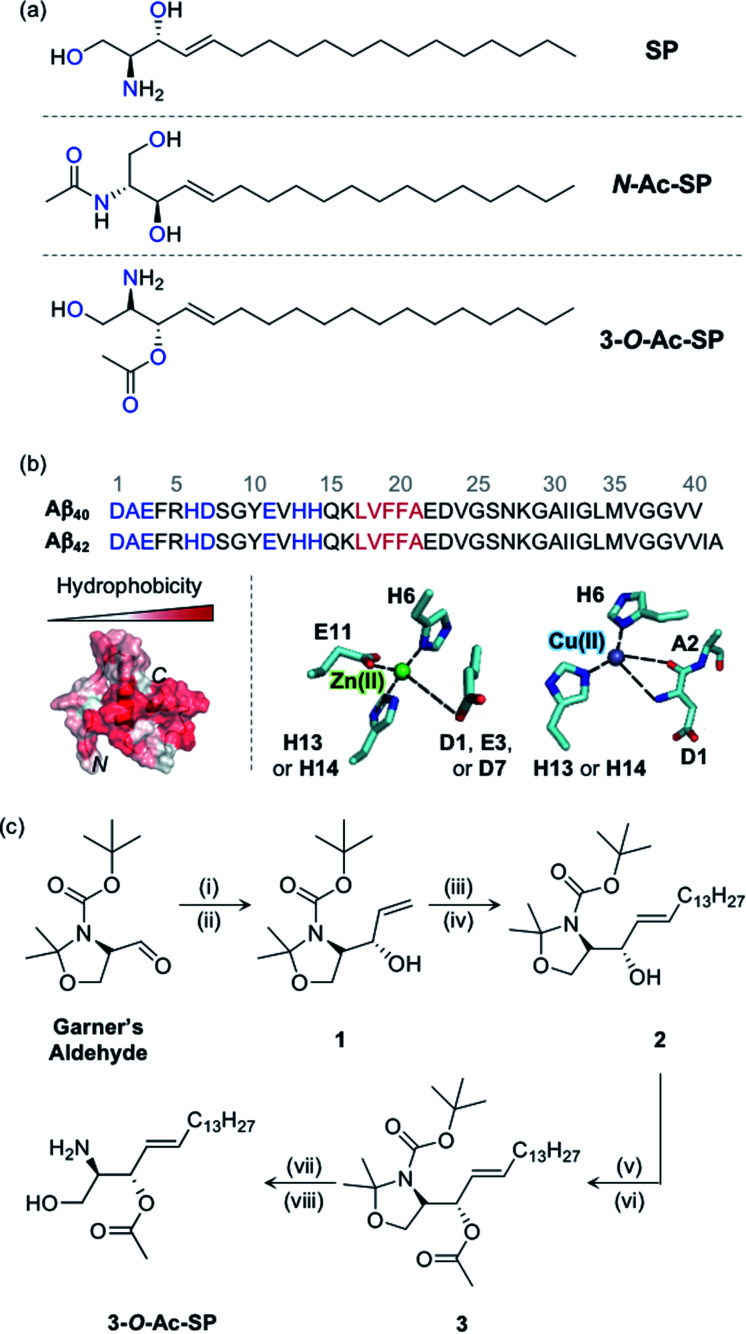
SP and acetylsphingosines studied in this work. (a) Chemical structures of SP, *N*-Ac-SP, and 3-*O*-Ac-SP. An acetyl group was incorporated into the structure of SP at the *N*- or 3-*O*-position yielding *N*-Ac-SP and 3-*O*-Ac-SP, respectively. SP, (2*S*,3*R*)-2-aminooctadec-4-*trans*-ene-1,3-diol; *N*-Ac-SP, (2*S*,3*R*,4*E*)-2-(acetylamino)-4-octadecene-1,3-diol; 3-*O*-Ac-SP, (2*S*,3*S*,*E*)-2-amino-1-hydroxyoctadec-4-en-3-yl acetate. Potential donor atoms for metal binding are highlighted in blue. (b) Amino acid sequences of Aβ_40_ and Aβ_42_ (top) as well as hydrophobicity and metal binding of Aβ (bottom). Top: the amino acid residues involved in metal coordination and the self-recognition site are highlighted in blue and red, respectively. Bottom: (left) structure of Aβ_40_ (PDB ID: 2LFM)^35^ exhibiting a degree of hydrophobicity; (right) examples of Zn(ii) and Cu(ii) coordination to Aβ.^30,31^ Possible fifth ligands on the metal centers are not shown in this figure. (c) Synthetic routes to 3-*O*-Ac-SP. Reagents and conditions: (i) (1) vinylmagnesium bromide, THF, −30 °C (30 min) to 0 °C (1 h); (ii) (2) sat. NH_4_Cl (aq); (iii) 1-pentadecene, second-generation Grubbs catalyst; (iv) CH_2_Cl_2_, room temperature, 16 h; (v) acetyl chloride, 4-dimethylaminopyridine; (vi) pyridine : CH_2_Cl_2_ (2 : 1), room temperature, overnight; (vii) trifluoroacetic acid; (viii) CH_2_Cl_2_ : H_2_O (2 : 1), room temperature, overnight.

As illustrated in Fig. 1a, sphingosine (SP) is an amino alcohol with an unsaturated hydrocarbon chain presenting the structural backbone of sphingolipids.^5,11,12^ Amphiphilic compounds, including SP, are known to form micelles and micellar aggregates at concentrations above their critical aggregation concentrations (CACs).^13–17^ Several reports suggest the subcellular localization of SP in the plasma membrane, cytosol, lysosome, mitochondria, Golgi apparatus, and endoplasmic reticulum.^7,18,19^ As a component of cellular regulation circuits, SP is engaged in regulating the release of neurotransmitters by controlling pre-synaptic vesicle fusion under normal conditions.^20,21^ In addition, pathological shifts in the sphingolipid rheostat manifesting increased levels of SP are associated with pro-apoptotic conditions contributing towards neurodegeneration.^6,21,22^ Recently, elevated concentrations of SP have been detected in the post-mortem brains of Alzheimer's disease (AD) patients.^22^ Despite such notions, a connection between SP and AD is only beginning to be revealed.

AD is the most common neurodegenerative disease exhibiting significant intricacy in its pathology involving several pathogenic features, such as proteopathy and metal ion dyshomeostasis.^23–34^ The proteopathic implications of AD include the pathology of amyloid-β (Aβ), while the metal ion hypothesis focuses on the dualistic pathogenic qualities of loss-of-function and gain-of-toxicity through metal ion dyshomeostasis and miscompartmentalization.^23–34^ Increasing evidence indicates the independent and synergistic contributions of Aβ and metal ions towards neurodegeneration.^23–34^ Aβ, the main component of senile plaques, is an aggregation-prone peptide that forms neurotoxic species.^24–35^ Metal ions [*e.g.*, Zn(ii) and Cu(ii)] colocalized with Aβ aggregates in senile plaques can coordinate to Aβ peptides, as displayed in Fig. 1b, and affect the aggregation of Aβ.^24–34^ The contemporary viewpoint on the complex pathology of AD considers the inter-relationships among different pathological elements found in the disease.^24,25,32^

In this study, SP was hypothesized to interact with Aβ and metal ions and subsequently alter the aggregation and cytotoxicity of metal-free Aβ and metal-bound Aβ (metal–Aβ). Such interactions under pathological conditions are supported by four fundamental aspects: (i) potential points of convergence at the subcellular level for SP, Aβ, and metal ions,^7,18,19,36^ (ii) enhanced concentrations of SP, Aβ, and metal ions in AD-affected brains,^22,24–26^ (iii) amphiphilic properties of both SP and Aβ, and (iv) inclusion of potential metal-binding sites on the structure of SP. Three sphingolipids, including SP, *N*-acetylsphingosine (*N*-Ac-SP), and 3-*O*-acetylsphingosine (3-*O*-Ac-SP) shown in Fig. 1a, were utilized to experimentally confirm these interactions at the molecular level in association with their ability to form micelles and micellar aggregates and evaluate their impact on the aggregation and cytotoxicity of both metal-free Aβ and metal–Aβ. Overall, we provide a multidisciplinary perspective on the interactions and pathogenic connections between our molecular subjects.

## Results and discussion

### Preparation and properties of SP and acetylsphingosines

SP, a skeletal structure of sphingolipids, is subject to a diverse range of structural variations such as acylation, phosphorylation, and glycosylation, often serving as a measure to control cellular signaling.^5,12,37^ In particular, acylation is a universal modification of biomolecules with nitrogen (N) and oxygen (O) atoms generating amide and ester groups, respectively.^38^ In this work, we incorporated the acetyl functionality, as the simplest acyl group, onto the structure of SP to construct *N*-Ac-SP and 3-*O*-Ac-SP, as presented in Fig. 1a, and employed them as representative sphingolipids, along with SP. SP and *N*-Ac-SP were obtained commercially, while 3-*O*-Ac-SP was synthesized following previously reported procedures with slight modifications, as summarized in Fig. 1c.^39–43^ The Grignard reaction of Garner's aldehyde with vinylmagnesium bromide produced 1. Next, 2 was generated *via* the cross-metathesis reaction of 1 with 1-pentadecene using the second-generation Grubbs' catalyst. The subsequent acetylation of 2 with acetyl chloride under basic conditions afforded 3. Lastly, after acid-catalyzed deprotection of the cyclic *N*,*O*-aminal and *tert*-butoxycarbonyl group in 3, the final product, 3-*O*-Ac-SP, was obtained in a modest yield (Fig. S1†).

SP and the two acetylsphingosines are expected to interact with Aβ and metal ions based on their molecular structures depicted in Fig. 1a. For Aβ interaction, the alkenyl chains and the amino alcohol moieties with or without acetyl substitution of these molecules may interact with the hydrophobic and hydrophilic regions of Aβ, respectively, as presented in Fig. 1b.^26^ All three compounds embody potential metal-binding sites. SP has two bidentate sites for metal ions, *i.e.*, the moieties of 2-aminoethanol and 1,3-propanediol. The two acetylsphingosines also have possible metal-binding sites composed of N and O donor atoms. Moreover, the amphiphilicity of these compounds drives their self-aggregation into micelles and micellar aggregates at concentrations above their CACs.^13–17^ Their corresponding CACs were measured by dynamic light scattering in appropriate buffered solutions. The CACs of SP, *N*-Ac-SP, and 3-*O*-Ac-SP were determined to be *ca.* 3–81 μM, 7–27 μM, and 29–68 μM, respectively, depending on pH and the presence of a salt (*e.g.*, NaCl) (Fig. S2†). The aggregation is initiated at their corresponding CACs. The concentration ratio of micelles and micellar aggregates to monomers theoretically increases in a manner proportional to their concentrations above the CACs.^44,45^

### Interactions of SP and acetylsphingosines with Aβ

**Fig. 2:**
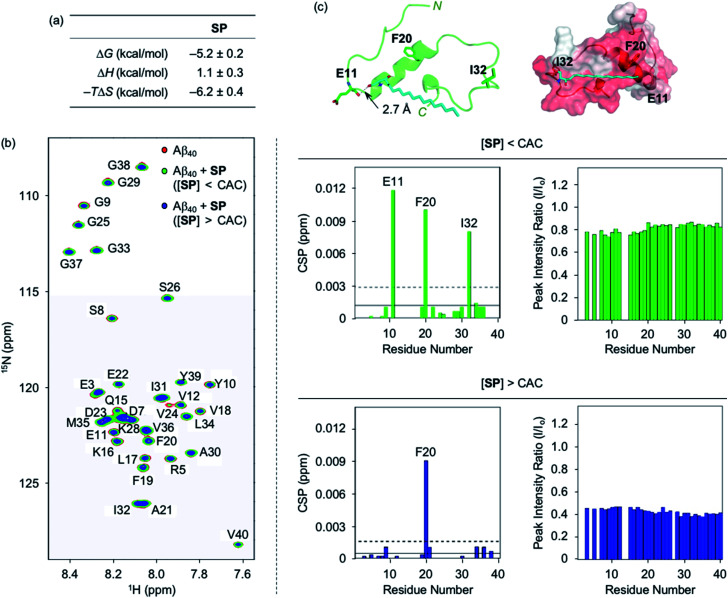
Interactions of SP with Aβ. (a) Thermodynamic parameters for binding of SP to Aβ_40_ determined by ITC. ITC data are shown in Fig. S3.† Conditions: [Aβ_40_] = 500 μM; [SP] = 200 μM (1% v/v DMSO); 20 mM HEPES, pH 7.4; 10 °C. (b) 2D ^1^H–^15^N SOFAST-HMQC NMR analysis of the samples containing SP and ^15^N-labeled Aβ_40_. The spectra (left), CSPs (middle), and peak intensity ratios (right) of ^15^N-labeled Aβ_40_ were acquired upon addition of SP at the concentrations below or above the CAC. Two horizontal lines represent the average chemical shift (solid line) plus one standard deviation (dashed line). Conditions: [^15^N-labeled Aβ_40_] = 40 μM; [SP] = 2 μM (below the CAC) or 100 μM (above the CAC) (1% v/v DMSO); 20 mM HEPES, pH 7.4; 10% v/v D_2_O; 10 °C. (c) Possible interactions between SP (cyan) and Aβ_40_ monomer (PDB ID: 2LFM)^35^ visualized by docking studies [cartoon (left) and surface (right) versions]. The amino acid residues in Aβ_40_ with relatively large CSPs observed from the NMR studies are depicted in stick representation (left). The dashed line indicates possible hydrogen bonding between SP and Aβ_40_ monomer within 3.0 Å. Hydrophobic contacts of SP onto the self-recognition site and the *C*-terminal region of Aβ_40_ monomer are observed (right). Hydrophilic to hydrophobic amino acid residues are indicated in a gradient from white to red.

To analyze the molecular-level interactions between SP, *N*-Ac-SP, or 3-*O*-Ac-SP and Aβ, binding studies were conducted by isothermal titration calorimetry (ITC) and two-dimensional band selective optimized flip-angle short transient-heteronuclear multiple quantum correlation nuclear magnetic resonance (2D SOFAST-HMQC NMR) spectroscopy. To avoid undesired aggregation of Aβ during experiments, we employed Aβ_40_ that has a lower aggregation propensity than Aβ_42_.^26,29,32,33^ The concentration greater than the compounds' CACs was used for ITC experiments. Under these conditions, changes in heat were measured upon titrating Aβ_40_ into the solutions of SP, *N*-Ac-SP, and 3-*O*-Ac-SP to avoid their conformational transformations from the mixture of micelles and micellar aggregates to monomers that can induce heat changes during the titrations.^46^ For the association of SP with Aβ, an endothermic isotherm was recorded with negative Gibbs free energy change (Δ*G*) displaying the spontaneous complex formation with an energetically unfavorable enthalpic contribution [*i.e.*, positive enthalpy change (Δ*H*)], as illustrated in Fig. 2a and S3.† The data suggested that interactions between the titrand and the titrant were thermodynamically favored by positive entropy change (Δ*S*) and could be mainly driven by hydrophobic interactions based on the greater contribution of entropy over enthalpy. It should be noted that the dissociation constant (*K*_d_) of SP for Aβ could not be accurately determined due to its heterogeneous population in solution, including monomers, micelles, and micellar aggregates.

Moving forward, the interactions between SP and Aβ were further probed in detail by 2D SOFAST-HMQC NMR spectroscopy. As depicted in Fig. 2b, chemical shift perturbations (CSPs) were monitored when ^15^N-labeled Aβ_40_ was incubated with SP at concentrations below and above the CAC. The SP-induced CSPs for ^15^N-labeled Aβ_40_ were mostly moderate in magnitude suggesting weak and nonspecific interactions between the compound and Aβ. The E11, F20, I32, and F20 residues were subject to greater CSPs by treatment of the compound at concentrations below and above the CAC, respectively. Furthermore, the intensity of all amino acid residues was reduced upon the addition of SP to ^15^N-labeled Aβ_40_, which implies that SP may mediate the production of NMR-invisible Aβ aggregates.^47^ Possible interactions between SP and the amino acid residues in Aβ_40_ presenting relatively significant CSPs were visualized by docking studies employing the monomeric structure of Aβ_40_ (PDB ID: 2LFM) that was identified by NMR in aqueous solution.^35^ As shown in Fig. 2c, SP was simulated to form hydrogen bonding (*i.e.*, an O–H moiety of SP with the backbone carbonyl group between E11 and V12) and be positioned near the hydrophobic region of Aβ. The end of SP's hydrocarbon chain was positioned near the self-recognition site responsible for driving Aβ aggregation.^24,29^

**Fig. 3:**
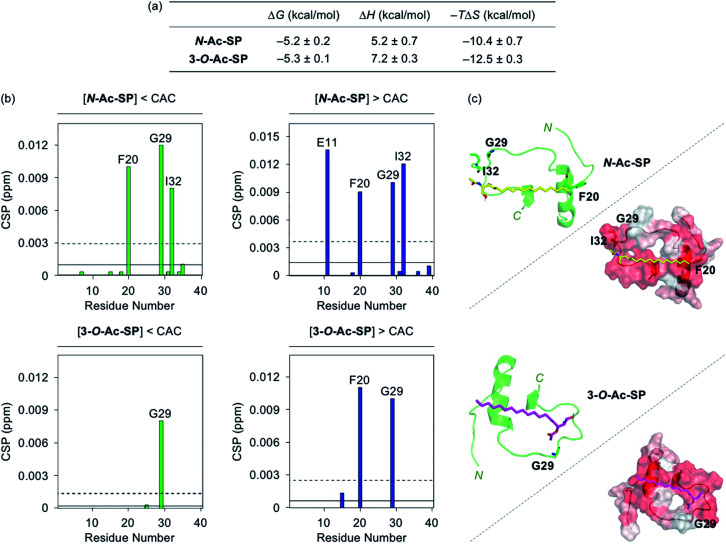
Interactions of *N*-Ac-SP and 3-*O*-Ac-SP with Aβ monomer. (a) Thermodynamic parameters for binding of *N*-Ac-SP and 3-*O*-Ac-SP to Aβ_40_ measured by ITC. ITC data are shown in Fig. S4.† Conditions: [Aβ_40_] = 500 μM; [acetylsphingosine] = 200 μM (1% v/v DMSO); 20 mM HEPES, pH 7.4; 10 °C. (b) CSPs in Aβ_40_ upon addition of *N*-Ac-SP and 3-*O*-Ac-SP at the concentrations below or above their CACs obtained from 2D ^1^H–^15^N SOFAST-HMQC NMR spectra (Fig. S5†). Average CSPs (solid line) are indicated with standard deviations (dashed line). Conditions: [^15^N-labeled Aβ_40_] = 40 μM; [compound] = 2 μM (below the CAC) or 100 μM (above the CAC) (1% v/v DMSO); 20 mM HEPES, pH 7.4; 10% v/v D_2_O; 10 °C. (c) Possible conformations of *N*-Ac-SP (yellow) and 3-*O*-Ac-SP (pink) docked with Aβ_40_ monomer (PDB ID: 2LFM)^35^ [cartoon (left) and surface (right) versions]. The amino acid residues in Aβ_40_ with relatively large CSPs are presented in stick representation (left). Hydrophobic contacts of the acetylsphingosines onto the self-recognition site and the *C*-terminal region of Aβ_40_ monomer are displayed (right). Hydrophilic to hydrophobic amino acid residues are indicated in a gradient from white to red.

Interactions between the two acetylsphingosines and Aβ exhibited spontaneous complexation showing an endothermic process with the preferential entropic contributions compensating the unfavorable enthalpic contributions, as displayed in Fig. 3a and S4.† In a manner similar to SP, the interactions of *N*-Ac-SP and 3-*O*-Ac-SP with Aβ may be primarily directed by hydrophobic interactions. Interestingly, the magnitude of entropic contributions seemed to increase in the order of SP, *N*-Ac-SP, and 3-*O*-Ac-SP, while that of enthalpic contributions was in reverse order. This may suggest that the acetylsphingosines are more involved in the exclusion of water molecules from the surface of Aβ, relative to SP, facilitating their hydrophobic interactions with Aβ. It should be noted that the accurate *K*_d_ values of the acetylsphingosines for Aβ were not able to be obtained due to their heterogeneous conformations in solution at concentrations above the CACs. Based on the comparable Δ*G* values for the association of SP or the acetylsphingosines towards Aβ, which are logarithmically related to their *K*_d_ values, their binding affinities for Aβ are speculated to be in a similar range. As presented in Fig. 3b and S5,† 2D ^1^H–^15^N SOFAST-HMQC NMR studies revealed relatively low but detectable CSPs of several amino acid residues in ^15^N-labeled Aβ_40_ upon incubation with the acetylsphingosines (*e.g.*, F20, G29, and I32 for *N*-Ac-SP and G29 for 3-*O*-Ac-SP at the concentrations below the CACs; E11, F20, G29, and I32 for *N*-Ac-SP and F20 and G29 for 3-*O*-Ac-SP at the concentrations above the CACs). These observations indicate weak and nonspecific interactions of both *N*-Ac-SP and 3-*O*-Ac-SP towards Aβ. No notable decrease in the overall peak intensity of ^15^N-labeled Aβ_40_ was observed by treatment of the acetylsphingosines. Moreover, docking studies indicated that both acetylsphingosines were situated on the hydrophobic region of Aβ_40_ pertaining to the amino acid residues that exhibited relatively significant CSPs from 2D NMR studies, as shown in Fig. 3c. The hydrophobic interactions at the self-recognition site of Aβ_40_ were visualized with the ends of hydrocarbon chains of the two acetylsphingosines. Taken together, the ITC and 2D NMR studies demonstrate that SP and the acetylsphingosines, in either their monomeric or micellar forms, can directly interact with Aβ, with the support of docking studies.

### Metal-binding properties of SP and acetylsphingosines in the absence and presence of Aβ

**Fig. 4:**
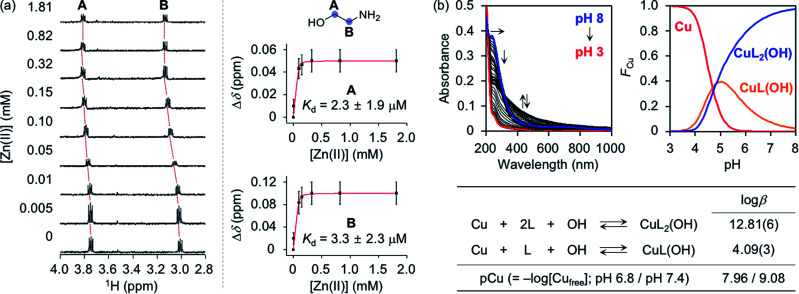
Metal-binding properties of 2-aminoethanol. (a) ^1^H NMR spectra (left) and changes in the chemical shifts (Δ*δ*; right) of 2-aminoethanol with various concentrations of Zn(ii). Red curves were fitted to the Δ*δ* values of the peaks assigned to the protons at the A and B positions to estimate a binding affinity of the compound for Zn(ii). Error bars indicate the standard error from three independent experiments. Conditions: [2-aminoethanol] = 100 μM; [Zn(ii)] = 0.005, 0.01, 0.05, 0.10, 0.15, 0.32, 0.82, and 1.81 mM; [DSS (2,2-dimethyl-2-silapentane-5-sulfonate sodium salt)] = 10 μM (internal reference); H_2_O : D_2_O (9 : 1). (b) Solution speciation studies of Cu(ii)–2-aminoethanol (L) complexes. Top: UV-vis variable-pH titration spectra (left) and solution speciation diagram (right) were obtained by spectrophotometric titrations of Cu(ii)–ligand complexes (*F*_Cu_ = fraction of species at given pH). Bottom: the values of log *β* and pCu of Cu(ii)–ligand complexes were summarized in the table. The errors in the last digit are shown in parentheses. Charges are omitted for clarity. Conditions: [2-aminoethanol] = 400 μM; [Cu(ii)] = 200 μM; room temperature; *I* = 0.10 M NaCl.

To assess whether SP, *N*-Ac-SP, and 3-*O*-Ac-SP can bind to metal ions in aqueous media, titration experiments with Zn(ii) and Cu(ii) were performed using ^1^H NMR and UV-visible (UV-vis) spectroscopy, respectively. It should be noted that potential metal-binding moieties in the structure of SP, *i.e.*, 2-aminoethanol and 1,3-propanediol, were used for these studies due to its limited aqueous solubility.^48^ When Zn(ii) was added to a solution of 2-aminoethanol, the peaks assigned to the α-protons became deshielded in the NMR spectra, as illustrated in Fig. 4a. This observation could be explained by the lower electron density of 2-aminoethanol upon binding to Zn(ii). Moreover, our titration measurements further estimated the *K*_d_ value of SP towards Zn(ii) to be in the micromolar range under our experimental conditions. In the case of 1,3-propanediol, the *K*_d_ value for Zn(ii) could not be determined although excess amounts of Zn(ii) were titrated into the solution of the ligand (data not shown). As expected from the hard and soft acid and base concept^49^ and the size of chelate rings,^50^ Zn(ii) chelation of 2-aminoethanol, with N and O donor atoms forming a five-membered chelate ring, was more favorable than that of 1,3-propanediol containing two O donor atoms to generate a six-membered chelate ring. Thus, Zn(ii) binding of SP can occur preferentially *via* the 2-aminoethanol moiety. The metal-binding properties of the acetylsphingosines were also considered with their potential metal-binding sites (*i.e.*, 1,3-propanediol for *N*-Ac-SP; 2-aminoethanol for 3-*O*-Ac-SP shown in Fig. 1a). *N*-Ac-SP includes the *N*-(2-hydroxyethyl)acetamide group as an additional site for possible metal binding. Unfortunately, the *K*_d_ value of this moiety for Zn(ii) could not be obtained even with excess concentrations of Zn(ii) (data not shown). Therefore, SP and 3-*O*-Ac-SP exhibit a greater Zn(ii)-binding ability than *N*-Ac-SP through the 2-aminoethanol group.

Cu(ii)-binding properties of 2-aminoethanol were analyzed *via* solution speciation studies with UV-vis variable-pH titrations. Spectrophotometric titrations of 2-aminoethanol (L) in the absence of Cu(ii) were first performed to estimate its acidity constant (p*K*_a_), as shown in Fig. S6.† The solution speciation diagram depicts the presence of neutral (L) and monoprotonated (LH) forms of 2-aminoethanol in the pH range from 3 to 11. At pH 6.8, a condition plausibly representing a physiologically acidotic environment where Aβ aggregation is suggested to be facilitated in the presence of Cu(ii),^51^ and at physiological pH (pH 7.4), the LH form of the ligand was predicted to be major. Employing the p*K*_a_ value [9.79(0)] of the ligand, solution speciation experiments in the presence of Cu(ii) were further carried out to determine the Cu(ii)-to-ligand stoichiometry and its binding affinity for Cu(ii). As shown in Fig. 4b, Cu(ii)–ligand complexes with 1 : 1 and/or 1 : 2 Cu(ii)-to-ligand stoichiometry were indicated in the solution speciation diagram at pH 6.8 and pH 7.4. Moreover, the binding affinity of the ligand for Cu(ii) was predicted based on the values of stability constants (log *β*) and pCu [pCu = −log[Cu_free_], where [Cu_free_] indicates the concentration of unchelated Cu(ii)]. Considering the protonation and complexation for 2-aminoethanol at the given pH and concentrations of the ligand and Cu(ii), the value of pCu was obtained as indicative of the relative Cu(ii)-binding ability of the ligand under experimental conditions.^52,53^ 2-Aminoethanol exhibited the pCu values as 7.96 and 9.08 at pH 6.8 and pH 7.4, respectively, that are expected to be higher than pZn at each pH based on Irving–Williams series.^54^ According to previously reported studies, Cu(ii) in the solution of aminosugars and aminoglycosides containing aminoalcohol moieties has been observed to lower the p*K*_a_ of hydroxyl groups in the ligands, corroborating the higher binding affinity of 2-aminoethanol for Cu(ii) than Zn(ii).^55–57^ It should be noted that the values of pCu for the 1,3-propanediol and *N*-(2-hydroxyethyl)acetamide moieties could not be obtained due to their insignificant optical changes upon addition of Cu(ii) (data not shown). Overall, SP and 3-*O*-Ac-SP can coordinate to Cu(ii) through the 2-aminoethanol functionality. The comparable metal-binding affinities of 2-aminoethanol in SP and 3-*O*-Ac-SP with those of Aβ [*K*_d_ = 10^−9^ to 10^−6^ M for Zn(ii)–Aβ at pH 7.4; *K*_d_ = 10^−6^ to 10^−12^ M and pCu = *ca.* 6–12 for Cu(ii)–Aβ in the pH range from 6.5 to 7.4]^24,58^ support their ability to competitively bind to metal ions with Aβ.

**Fig. 5:**
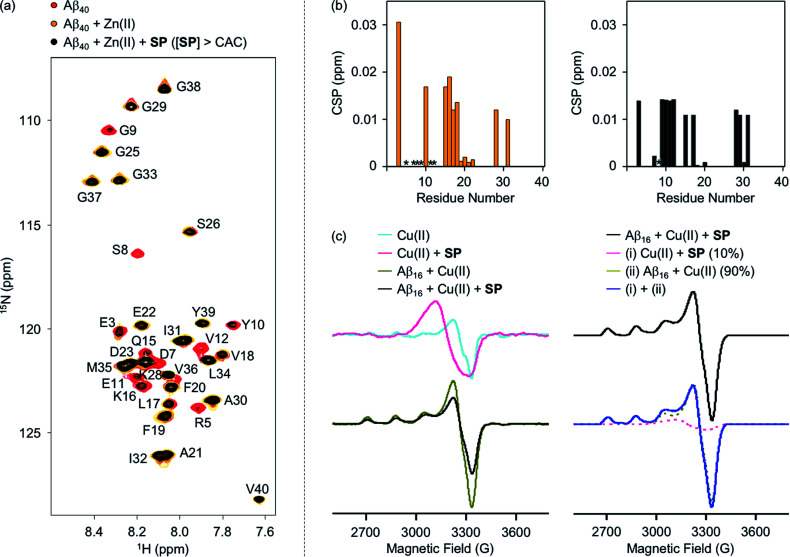
Interactions of SP with metal–Aβ analyzed by 2D ^1^H–^15^N SOFAST-HMQC NMR and EPR spectroscopy. (a) 2D NMR spectra of ^15^N-labeled Aβ_40_ with and without Zn(ii) and SP at the concentration above the CAC. (b) CSPs of the Zn(ii) samples in the absence (left) and presence (right) of SP at the concentration above the CAC. Upon addition of Zn(ii) to the solution of ^15^N-labeled Aβ_40_ with and without SP, the NMR signals of the amino acid residues indicated in asterisks (*) disappeared. Conditions: [^15^N-labeled Aβ_40_] = 40 μM; [Zn(ii)] = 40 μM; [SP] = 100 μM (above the CAC) (1% v/v DMSO); 20 mM HEPES, pH 7.4; 10% v/v D_2_O; 10 °C. (c) EPR spectra of the Cu(ii) samples with and without SP and Aβ_16_. The simulated spectrum (blue), similar to the normalized experimental spectrum of the Cu(ii) sample with Aβ_16_ and SP (black), was obtained by weighted summation of the normalized spectra of the Cu(ii) samples with either SP (10%; pink) or Aβ_16_ (90%; dark yellow). Conditions: [Aβ_16_] = 100 μM; [Cu(ii)] = 100 μM; [SP] = 400 μM (above the CAC) (1% v/v DMSO); 20 mM HEPES, pH 6.8, 150 mM NaCl; 37 °C; 24 h; constant agitation. The EPR spectra were collected with the following experimental parameters: microwave frequency, 9.41 GHz; microwave power, 2 mW; modulation frequency, 100 kHz; modulation amplitude, 10 G; time constant, 0.01 ms; conversion time, 60 ms; sweep time, 120 s; number of scan, 4; temperature, 100 K.

To evaluate whether SP containing the 2-aminoethanol group can chelate out the metal ion from metal–Aβ, its interaction with metal–Aβ was analyzed by 2D SOFAST-HMQC NMR and electron paramagnetic resonance (EPR) spectroscopy (Fig. 5, S7, and S8†). As depicted in Fig. 5b, the addition of Zn(ii) into the solution of ^15^N-labeled Aβ_40_ led to remarkable changes in the NMR signals of the amino acid residues in the *N*-terminal region. Particularly, the peaks of the amino acid residues close to the Zn(ii)-binding site in Aβ (Fig. 1b), *e.g.*, R5, D7, S8, G9, E11, and V12, disappeared, indicative of Zn(ii) binding to Aβ.^59,60^ When SP at the concentration above its CAC was introduced into the solution of ^15^N-labeled Aβ_40_ in the presence of Zn(ii), the peak signals corresponding to most of the aforementioned amino acid residues were restored to certain extents. This suggests that Zn(ii) binding to Aβ could be partially disrupted by SP. *N*-Ac-SP, which does not include the 2-aminoethanol moiety, was not able to affect binding of Zn(ii) to Aβ at the concentration above its CAC (Fig. S9†), as expected from its relatively poor metal-binding ability. It should be noted that the ability of the compounds at concentrations below their CACs to chelate out Zn(ii) from Zn(ii)–Aβ could not be distinguishably detected under our experimental conditions (*i.e.*, [^15^N-labeled Aβ_40_] = 40 μM; [compound] = 2 μM), as presented in Fig. S10 and S11.† Overall, SP with its micellar species potentially sequesters Zn(ii) from Zn(ii)–Aβ, which is expected based on the Zn(ii)-binding affinity of 2-aminoethanol in SP (Fig. 4a).

The interaction of SP with Cu(ii)–Aβ at the concentration above its CAC was examined by EPR spectroscopy. For the EPR studies, Aβ_16_, an *N*-terminal fragment of full-length Aβ that binds to metal ions and shows no significant aggregation,^24^ was used. Upon treatment of SP into the solution of Cu(ii), the shifts in symmetry of the *g*- and *A*-values were observed from axial to isotropic, as depicted in Fig. 5c and S8,† suggesting its binding to Cu(ii). This is expected from the Cu(ii)-binding affinity of 2-aminoethanol (Fig. 4b). The sample of Cu(ii) with Aβ_16_ produced an axial EPR signal at *g*_⊥_ = 2.05 and *g*_‖_ = 2.28 with the hyperfine splitting of *A*_⊥_ = 6.96 G and *A*_‖_ = 167.75 G (Fig. 5c and S8†), indicative of the complexation between Cu(ii) and Aβ_16_.^61^ When SP was added into the solution containing Cu(ii) and Aβ_16_, the EPR spectrum was different from that of the Cu(ii) samples with either SP or Aβ_16_. Cu(ii) binding was further quantified by weighted summation of the normalized spectra of the Cu(ii) samples containing either Aβ_16_ or SP following the previously reported procedure.^62–64^ As a result, the EPR spectrum of the Cu(ii) sample treated with both Aβ_16_ and SP exhibited a mixture of Cu(ii) bound to SP (10%) and Aβ_16_ (90%). Thus, the EPR studies demonstrate the ability of SP to potentially chelate out Cu(ii) from Cu(ii)–Aβ. In contrast, the EPR spectra for the Cu(ii) samples with *N*-Ac-SP in the absence and presence of Aβ_16_ were almost identical with those of compound-free Cu(ii) samples with and without Aβ_16_ (Fig. S8 and S12†), indicating that this compound cannot significantly affect Cu(ii) coordination to Aβ. These distinct behaviors of the compounds against metal–Aβ at the concentrations above their CACs are also supported by the previous studies displaying that ionic micelles can induce structural changes of peptides and proteins in a manner affecting their Cu(ii)-binding properties.^65–67^ Taken together, as expected from the compounds' metal-binding affinities, the spectroscopic studies manifest that SP potentially sequesters the metal ion from metal–Aβ.

### Influence of SP and acetylsphingosines on the aggregation of metal-free and metal-associated Aβ

**Fig. 6:**
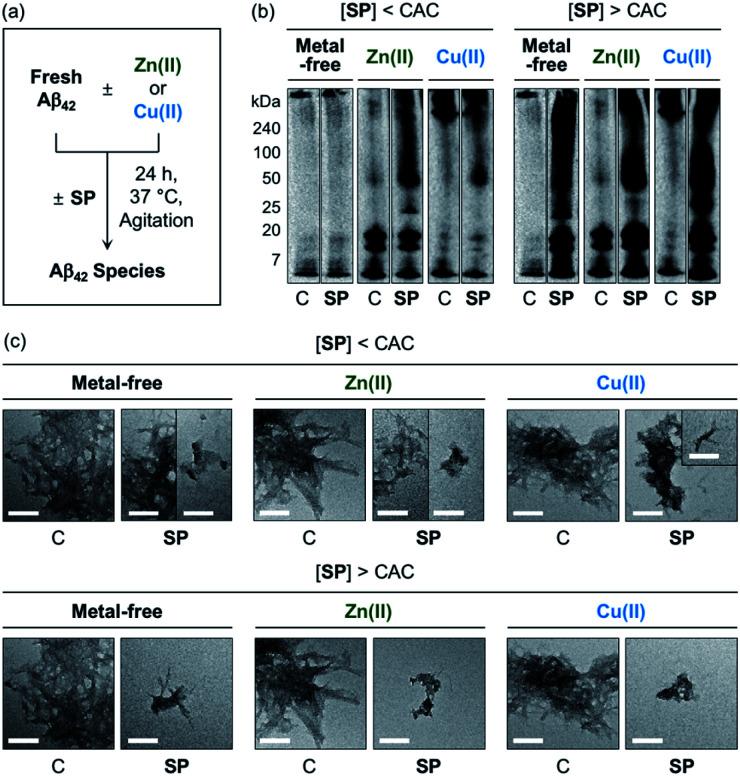
Impact of SP on metal-free and metal-induced Aβ_42_ aggregation. (a) Scheme of Aβ_42_ aggregation experiments. (b) Analysis of the size distribution of the resultant Aβ_42_ species by gel/Western blot using an anti-Aβ antibody (6E10). Lanes: (C) Aβ_42_ ± Zn(ii) or Cu(ii); (SP) C + SP. The original gel images are shown in Fig. S13.† (c) Morphologies of the resultant Aβ_42_ aggregates from (b) detected by TEM. Conditions: [Aβ_42_] = 25 μM; [Zn(ii) or Cu(ii)] = 25 μM; [compound] = 10 μM (below the CAC) or 100 μM (above the CAC) (1% v/v DMSO); 20 mM HEPES, pH 7.4 [for metal-free and Zn(ii)-containing samples] or pH 6.8 [for Cu(ii)-containing samples], 150 mM NaCl; 37 °C; 24 h; constant agitation. Scale bars = 200 nm.

The effects of SP, *N*-Ac-SP, and 3-*O*-Ac-SP, at concentrations below and above the CACs, on the aggregation of Aβ_40_ and Aβ_42_ with and without Zn(ii) and Cu(ii) were evaluated. Upon incubation of SP with freshly prepared metal-free and metal-treated Aβ, as displayed in Fig. 6, S14, and S15,† the size distribution and the morphology of the resultant Aβ species were analyzed by gel electrophoresis with western blotting (gel/Western blot) and transmission electron microscopy (TEM), respectively. Aβ species with molecular weights (MWs) in a range from *ca.* 4 kDa (monomer) to *ca.* 240 kDa (aggregate) can be probed as visible bands in gel/Western blot.^68^ In particular, the smearing bands from compound-treated Aβ samples, distinct from compound-free Aβ samples, indicate that the compound can vary Aβ aggregation. Aβ aggregates that are too large to penetrate the gel matrix can be visualized by TEM.

At the concentration below the CAC, SP did not significantly affect the aggregation of metal-free Aβ_42_, as shown in Fig. 6b. In the case of Zn(ii)–Aβ_42_ incubated with SP, however, more intense bands were detected at *ca.* 25 kDa with the MW range larger than *ca.* 50 kDa, relative to compound-untreated Zn(ii)–Aβ_42_. The sample of Cu(ii)–Aβ_42_ treated with SP indicated an increase in the levels of dodecameric or larger Aβ_42_ species (*ca.* ≥50 kDa), compared to that of compound-free Cu(ii)–Aβ_42_. Upon increasing the concentration of SP beyond the CAC, changes in the MW distributions of Aβ_42_ became more dramatic, showing smearing bands at *ca.* 4–240 kDa for metal-free Aβ_42_ and Cu(ii)–Aβ_42_. The intensity of the bands from the sample containing Zn(ii)–Aβ_42_ and SP was enhanced at *ca.* 4–20 kDa and above *ca.* 50 kDa.

In the TEM studies, as displayed in Fig. 6c, the incubation of SP with metal-free and metal-treated Aβ_42_ resulted in the generation of Aβ_42_ aggregates indicating a mixed morphology with both fibrillary and amorphous characteristics. The overall size of the aggregate species was reduced with the treatment of SP, compared to those of the compound-free samples. At the concentration above the CAC, the SP-induced changes in the sizes of Aβ_42_ aggregates became more prominent, forming remarkably smaller Aβ species. Based on these observations, SP was demonstrated to modulate the aggregation of both metal-free and metal-treated Aβ_42_ and such reactivities were further intensified with its micellar species. The results of gel/Western blot and TEM studies with metal-free Aβ_40_ and metal–Aβ_40_ showed lacking reactivity from the samples incubated with SP at the concentration below the CAC (Fig. S14†). At the concentration above the CAC, SP altered the aggregation of metal-free Aβ_40_ and Cu(ii)–Aβ_40_, but it did not significantly affect Zn(ii)–Aβ_40_ aggregation. As shown in the ITC and 2D NMR studies (*vide supra*), hydrophobic interactions between SP and Aβ may promote the effect of the compound on the formation of Aβ aggregates. The metal-binding properties of SP could further support its ability to change the aggregation of metal–Aβ. In particular, the capability of SP at concentrations above the CAC to partially sequester the metal ion from metal–Aβ is expected to contribute to its reactivity towards the aggregation of metal–Aβ. It is noteworthy that multiple interactions of SP with metal–Aβ at concentrations above the CAC are possible based on the observations and findings through the NMR and EPR studies. For example, the transient ternary complexation between metal–Aβ and SP and the interactions of metal-free and/or metal-bound SP with the hydrophobic core and *C*-terminal region of Aβ (Fig. S7†) could simultaneously occur, along with metal displacement by SP. These could result in the modulative impact of SP with its micellar species on the aggregation of metal–Aβ.

**Fig. 7:**
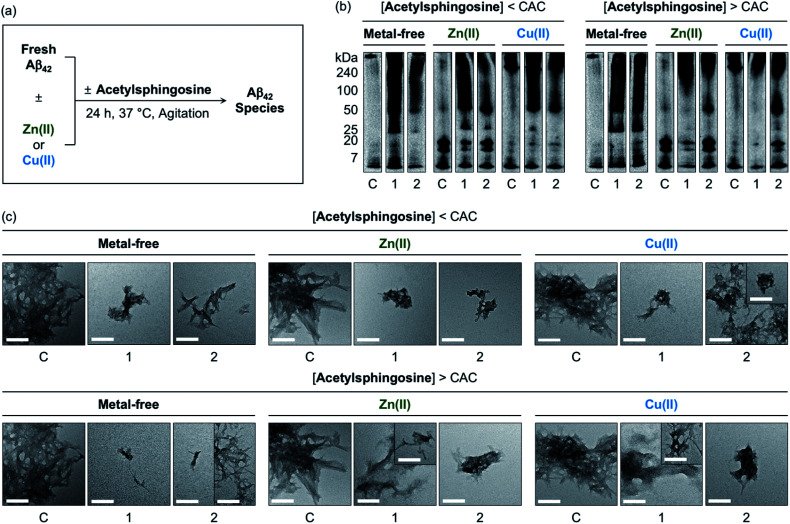
Influence of *N*-Ac-SP and 3-*O*-Ac-SP on the aggregation of metal-free Aβ_42_ and metal–Aβ_42_. (a) Scheme of Aβ_42_ aggregation experiments. (b) Analysis of the size distribution of the resultant Aβ_42_ species by gel/Western blot using an anti-Aβ antibody (6E10). Lanes: (C) Aβ_42_ ± Zn(ii) or Cu(ii); (1) C + *N*-Ac-SP; (2) C + 3-*O*-Ac-SP. The original gel images are shown in Fig. S13.† (c) Morphologies of the resultant Aβ_42_ aggregates from (b) detected by TEM. Conditions: [Aβ_42_] = 25 μM; [Zn(ii) or Cu(ii)] = 25 μM; [acetylsphingosine] = 10 μM (below the CAC) or 100 μM (above the CAC) (1% v/v DMSO); 20 mM HEPES, pH 7.4 [for metal-free and Zn(ii)-containing samples] or pH 6.8 [for Cu(ii)-containing samples], 150 mM NaCl; 37 °C; 24 h; constant agitation. Scale bars = 200 nm.

The generation of Aβ aggregates in the presence of *N*-Ac-SP and 3-*O*-Ac-SP was further analyzed (Fig. 7). At the concentrations under the CACs, *N*-Ac-SP produced new bands larger than *ca.* 25 kDa or 50 kDa for the samples of metal-free Aβ_42_ and metal–Aβ_42_, as depicted in Fig. 7b. When 3-*O*-Ac-SP was treated to metal-free Aβ_42_ and metal–Aβ_42_, the amount of aggregates with MW larger than *ca.* 50 kDa was increased, along with that of hexameric species at *ca.* 25 kDa, under metal-free and Zn(ii)-present conditions. At the concentrations above the CACs, *N*-Ac-SP noticeably varied the MW distributions larger than *ca.* 25 kDa for metal-free Aβ_42_, 7–25 kDa and above *ca.* 100 kDa for Zn(ii)–Aβ_42_, and 7–240 kDa for Cu(ii)–Aβ_42_. In the case of 3-*O*-Ac-SP, a new smearing band in the MW range larger than *ca.* 25 kDa was detected from metal-free Aβ_42_, while that with MW larger than *ca.* 50 kDa was observed for metal–Aβ_42_. In particular, the treatment of 3-*O*-Ac-SP with Cu(ii)–Aβ_42_ led to the detection of a band at *ca.* 25 kDa. The TEM studies revealed that both *N*-Ac-SP and 3-*O*-Ac-SP at the concentrations below their CACs fostered the production of smaller Aβ_42_ aggregates, compared to compound-untreated metal-free Aβ_42_ and metal–Aβ_42_, as presented in Fig. 7c. Upon addition of the acetylsphingosines at the concentrations above their CACs, relative to compound-free peptide samples, smaller aggregates of Aβ_42_ were exhibited with a mixture of morphologies with filamentous or amorphous qualities in the absence and presence of metal ions. As shown in Fig. S15,† the acetylsphingosines did not discernably influence the aggregation of metal-free and metal-treated Aβ_40_. Overall, SP, *N*-Ac-SP, and 3-*O*-Ac-SP are capable of modifying Aβ aggregation in the absence and presence of metal ions to distinct degrees at concentrations below and above their CACs.

### Impact of SP and acetylsphingosines on the toxicity induced by metal-free Aβ and metal–Aβ in living cells

**Fig. 8:**
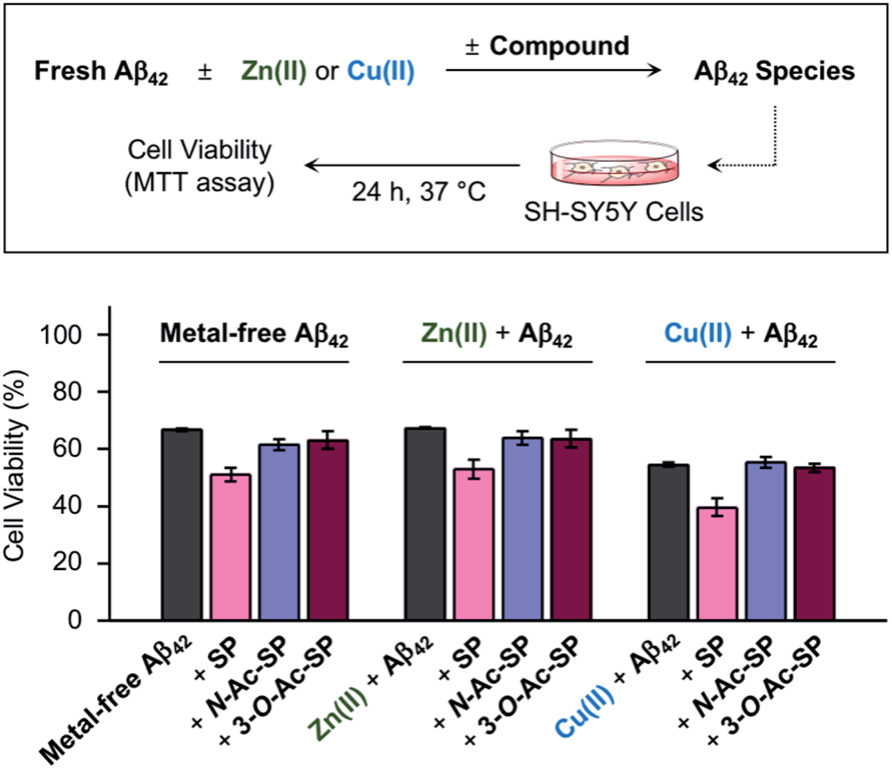
Effect of SP, *N*-Ac-SP, and 3-*O*-Ac-SP on the toxicity induced by metal-free Aβ_42_ or metal–Aβ_42_ in living cells. Cell viability, determined by the MTT assay, was calculated in comparison to that of the cells treated with an equivalent amount of the buffered solution with 0.2% v/v DMSO (20 mM HEPES, pH 7.4 or pH 6.8, 150 mM NaCl). Error bars indicate the standard error from three independent experiments. Conditions: [Aβ_42_] = 10 μM; [Zn(ii) or Cu(ii)] = 10 μM; [compound] = 10 μM (0.2% v/v DMSO); 20 mM HEPES, pH 7.4 [for metal-free and Zn(ii)-containing samples] or pH 6.8 [for Cu(ii)-containing samples], 150 mM NaCl.

Prior to cell studies with metal-free and metal-treated Aβ, the cytotoxicity of SP, *N*-Ac-SP, and 3-*O*-Ac-SP was investigated by the MTT assay employing human neuroblastoma SH-SY5Y (5Y) cells [MTT = 3-(4,5-dimethylthiazol-2-yl)-2,5-diphenyltetrazolium bromide]. The half-maximal inhibitory concentration (IC_50_) values for SP and *N*-Ac-SP were determined to be in the micromolar range, as summarized in Fig. S16a.† The IC_50_ value for 3-*O*-Ac-SP could not be obtained under our experimental conditions due to its relatively less toxicity (*ca.* >55% cell survival up to 100 μM). Moving forward, to examine the effects of SP and the acetylsphingosines on the cytotoxicity induced by Aβ_42_ in the absence and presence of metal ions, the samples containing Aβ with either Zn(ii) or Cu(ii), a compound, or both were treated to 5Y cells, as shown in Fig. 8. The treatment of SP with metal-free and metal-treated Aβ_42_ resulted in a further reduction in cell viability by *ca.* 15%. In contrast, when the cells were incubated with *N*-Ac-SP or 3-*O*-Ac-SP with metal-free and metal–Aβ_42_, the cell viabilities were not significantly affected. It should be noted that incubation of the compounds with 5Y cells at their concentrations used for cell studies with Aβ presented cell survival rates greater than *ca.* 80% in the absence and presence of Zn(ii) or Cu(ii) (Fig. S16b†). These results suggest that SP, but not *N*-Ac-SP and 3-*O*-Ac-SP, may have a deteriorative impact on the toxicity induced by metal-free and metal-treated Aβ_42_.

## Conclusions

As the lipid membrane-mediated perturbations in the properties of Aβ come to the fore, lipidomics is emerging as an aspect of AD prompting the advancement of our knowledge towards the relevancy of lipids in its pathology.^26,69–74^ AD-associated disturbances in the cerebral composition of sphingolipids, along with their neurobiological implications, suggest their potential roles in the progression of the disease.^21,22^ In this study, we first demonstrated that SP could bind to Aβ, mainly through hydrophobic interactions, and metal ions and consequently alter the aggregation of both metal-free Aβ and metal–Aβ. Moreover, the toxicity of metal-free Aβ and metal–Aβ in living cells was aggravated in the presence of SP. The acetylation of SP at *N*- and 3-*O*-positions resulted in more dominant hydrophobic interactions with Aβ, manifesting different degrees of the reactivity with metal-free Aβ and metal–Aβ. Additionally, the acetylsphingosines showed the diminished ability to vary the cytotoxicity of metal-free Aβ and metal–Aβ. Considering the aggregation of sphingolipids, we confirmed the distinct extents of modulative capacities towards the aggregation of metal-free Aβ_42_ and metal–Aβ_42_ depending on the presence of micellar SP or acetylsphingosines. Based on previously reported studies, micellar species can interact with Aβ in different manners from monomeric lipids (*e.g.*, the association of Aβ onto the surface of the membrane and the penetration of Aβ into the membrane).^69,73,75^ Further mechanistic studies in consideration of the charge state of the compounds and the curvature of their micellar species, along with the aggregation state and spatiotemporal distribution of Aβ, would assist in gaining a better understanding of molecular-level interactions of sphingolipids with Aβ at concentrations below and above their CACs.^69,73,75–78^ Collectively, this work illustrates that a single structural component of membranes, probably with its aggregated forms, can directly interact with the pathological components linked to AD and affect their properties and toxicity. Our overall findings can open new avenues for a molecular-level understanding of lipids with respect to their interactions and reactivities towards the pathological factors found in AD.

## Conflicts of interest

There are no conflicts to declare.

## Supplementary Material

SC-012-D0SC04366D-s001
