# Supplementary material for: Impact of sphingosine and acetylsphingosines on the aggregation and toxicity of metal-free and metal-treated amyloid-β
Source: Chem Sci. 2020 Dec 17;12(7):2456–66. doi: 10.1039/d0sc04366d (PMC8179336; doi:10.1039/d0sc04366d)
Supplement: SC-012-D0SC04366D-s001 [file SC-012-D0SC04366D-s001.pdf]

Electronic Supplementary Material (ESI) for Chemical Science.
This journal is © The Royal Society of Chemistry 2021

Supplementary Information

for

Impact of Sphingosine and Acetylsphingosines on the Aggregation and Toxicity of Metal-free and Metal-treated Amyloid-β

Yelim Yi,[a] Yuxi Lin,[‡b] Jiyeon Han,[‡a] Hyuck Jin Lee,[c] Nahye Park,[d] Geewoo Nam,[d] Young S. Park,[d] Young-Ho Lee*[befg] and Mi Hee Lim*[a]

[a]Department of Chemistry, Korea Advanced Institute of Science and Technology (KAIST), Daejeon 34141, Republic of Korea

[b]Research Center of Bioconvergence Analysis, Korea Basic Science Institute (KBSI), Ochang, Chungbuk 28119, Republic of Korea

[c]Department of Chemistry Education, Kongju National University, Gongju 32588, Republic of Korea

[d]Department of Chemistry, Ulsan National Institute of Science and Technology (UNIST), Ulsan 44919, Republic of Korea

[e]Research Headquarters, Korea Brain Research Institute, Daegu 41068, Republic of Korea

[f]Bio-Analytical Science, University of Science and Technology (UST), Daejeon 34113, Republic of Korea

[g]Graduate School of Analytical Science and Technology, Chungnam National University, Daejeon 34134, Republic of Korea

‡These authors contributed equally to this work.

*To whom correspondence should be addressed: miheelim@kaist.ac.kr and mr0505@kbsi.re.kr

Table of Contents

Experimental Section

Materials and Methods	S4
Synthesis of **3-*O*-Ac-SP**	S5
Critical Aggregation Concentrations (CACs)	S6
Isothermal Titration Calorimetry (ITC)	S6
Two-dimensional $^{1}H-^{15}N$ Band Selective Optimized Flip-angle Short Transient-Heteronuclear Multiple Quantum Correlation Nuclear Magnetic Resonance (2D $^{1}H-^{15}N$ SOFAST-HMQC NMR) Experiments	S7
Docking Studies	S7
Zn(II)-binding Studies	S8
Solution Speciation Studies	S8
Electron Paramagnetic Resonance (EPR) Measurements	S8
Aβ Aggregation Experiments	S9
Gel Electrophoresis with Western Blotting (Gel/Western Blot)	S9
Transmission Electron Microscopy (TEM)	S9
Cell Viability Studies	S10

**Fig. S1**	^{1}H and ^{13}C NMR spectra of **3-*O*-Ac-SP**	S11
**Fig. S2**	Aggregation of **SP** and the acetylsphingosines observed by DLS	S12
**Fig. S3**	ITC thermogram and binding isotherm of **SP** with $A\beta_{40}$ monomer	S13
**Fig. S4**	ITC thermograms and binding isotherms of ***N*-Ac-SP** and **3-*O*-Ac-SP** with $A\beta_{40}$ monomer	S14
**Fig. S5**	2D $^{1}H–^{15}N$ SOFAST-HMQC NMR spectra and peak intensity ratios of ^{15}N-labeled $A\beta_{40}$ upon treatment of ***N*-Ac-SP** or **3-*O*-Ac-SP** at the concentrations below or above their CACs	S15
**Fig. S6**	Solution speciation studies of 2-aminoethanol	S16
**Fig. S7**	Peak intensity ratios of ^{15}N-labeled $A\beta_{40}$ with Zn(II) in the absence and presence of **SP** at the concentration above the CAC obtained from the 2D $^{1}H–^{15}N$ SOFAST-HMQC NMR spectra	S17
**Fig. S8**	Experimental and simulated EPR spectra of the Cu(II) samples with and without $A\beta_{16}$, **SP**, and ***N*-Ac-SP**	S18
**Fig. S9**	2D $^{1}H–^{15}N$ SOFAST-HMQC NMR spectra of the samples containing	

^{15}N-labeled Aβ_{40}, Zn(II), and ***N*-Ac-SP** at the concentration above the CAC S19
Fig. S10 2D ^{1}H–^{15}N SOFAST-HMQC NMR analysis of the samples containing ^{15}N-labeled Aβ_{40}, Zn(II), and **SP** at the concentration below the CAC S20
Fig. S11 2D ^{1}H–^{15}N SOFAST-HMQC NMR analysis of the samples containing ^{15}N-labeled Aβ_{40}, Zn(II), and ***N*-Ac-SP** at the concentration below the CAC S21
Fig. S12 EPR spectra of the samples containing Cu(II) and Aβ_{16} in the absence and presence of ***N*-Ac-SP** S22
Fig. S13 Original gel images from Fig. 6, 7, S14, and S15 S23
Fig. S14 Impact of **SP** on metal-free and metal-induced Aβ_{40} aggregation S24
Fig. S15 Influence of ***N*-Ac-SP** and **3-*O*-Ac-SP** on the aggregation of metal-free Aβ_{40} and metal–Aβ_{40} S25
Fig. S16 Toxicity of **SP** and the acetylsphingosines in the absence and presence of metal ions in SH-SY5Y cells S26

References S27

Experimental Section

Materials and Methods. All chemical reagents were purchased from commercial suppliers and used as received unless otherwise stated. **SP** (purity 96%) and ***N*-Ac-SP** (purity 98%) were purchased from TCI Chemicals (Tokyo, Japan) and Merck KGaA (Darmstadt, Germany), respectively. ^{1}H and ^{13}C nuclear magnetic resonance (NMR) spectra of compounds were recorded on a Bruker AV400 NMR spectrometer [Bruker BioSpin, Rheinstetten, Germany; Department of Chemistry, KAIST, Daejeon, Republic of Korea]. High-resolution mass spectrometric analysis of compounds was carried out by micrOTOF-QII [Bruker Daltonics, Bremen, Germany; KAIST Analysis Center for Research Advancement (KARA), Daejeon, Republic of Korea]. Critical aggregation concentrations (CACs) of compounds were determined by a Malvern Zetasizer Nano ZSP DLS system (Malvern Panalytical Ltd., Worcestershire, UK). Optical spectra were recorded on an Agilent 8453 UV−visible (UV–vis) spectrophotometer (Santa Clara, CA, USA). Aβ_{16} (DAEF-RHDSGYEVHHQK), Aβ_{40} (DAEFRHDSGYEVHHQKLVFFAEDVGSNKGAIIGLMVGGVV), and Aβ_{42} (DAEFRHDSGYEVHHQKLVFFAEDVGSNKGAIIGLMVGGVVIA) were obtained from Peptide Institute, Inc. (Osaka, Japan). HEPES [2-(4-(2-hydroxyethyl)piperazin-1-yl)ethanesulfonic acid] was purchased from Sigma-Aldrich (St. Louis, MO, USA). The buffered solution was prepared in doubly distilled water [ddH$_2$O; a Milli-Q Direct 16 system (18.2 MΩ·cm; Merck KGaA)]. Trace metal contamination was removed from all solutions used for Aβ experiments by treating with Chelex (Sigma-Aldrich) overnight. Isothermal calorimetric titrations were performed by a PEAQ-ITC instrument (Malvern Panalytical Ltd.). 2D ^{1}H−^{15}N SOFAST-HMQC NMR spectra of uniformly ^{15}N-labeled Aβ_{40} (rPeptide, Georgia, GA, USA) were collected by a Bruker AVANCE II-800 MHz NMR spectrometer equipped with a cryoprobe [Bruker BioSpin; Korea Basic Science Institute (KBSI), Ochang, Republic of Korea]. Docking studies of compounds with Aβ_{40} monomer were carried out by AutoDock Vina 1.5.6 software. X-band continuous-wave (CW)-electron paramagnetic resonance (EPR) spectra were obtained by a Bruker EMXplus EPR spectrometer (Bruker BioSpin, Silberstreifen, Rheinstetten, Germany) equipped with an ER 4141VT digital temperature control system (Bruker BioSpin) and an ER 4119HS cavity (Bruker BioSpin). Images gained through gel/Western blot were visualized by a ChemiDoc MP imaging system (Bio-Rad, Hercules, CA, USA). Morphologies of larger Aβ aggregates produced from aggregation experiments were taken on a Tecnai F20 transmission electron microscope (FEI Company, Eindhoven, Netherlands; KARA, Daejeon, Republic of Korea). The human neuroblastoma SH-SY5Y cell line was purchased from the American Type Culture Collection (ATCC, Manassas, VA, USA). MTT [3-(4,5-dimethyl-2-thiazolyl)-2,5-diphenyl-2*H*-tetrazolium

bromide] was purchased from Sigma-Aldrich. Absorbance values for the MTT assay were determined by a SpectraMax M5e microplate reader (Molecular Devices, San Jose, CA, USA).

Synthesis of 3-*O*-Ac-SP [3-*O*-Acetylsphingosine, (*E*)-2-Amino-1-hydroxyoctadec-4-en-3-yl acetate]

1 [*tert*-Butyl (*S*)-4-((*S*)-1-hydroxyallyl)-2,2-dimethyloxazolidine-3-carboxylate]. **1** was synthesized following previously reported procedures with slight modifications.[1,2] Vinylmagnesium bromide [1.0 M in tetrahydrofuran (THF), 1.6 mL, 1.6 mmol] was added dropwise to Garner's aldehyde (0.17 mL, 0.78 mmol) in anhydrous THF (2.0 mL) at –30 °C for 30 min. The solution was gradually warmed to 0 °C for 1 h. The reaction was quenched with the saturated aqueous solution of NH_4Cl (2.0 mL) at –20 °C and then warmed to room temperature. After the mixture was extracted with ethyl acetate (EtOAc; 3x), the combined organic phase was washed with brine (1x) and dried over anhydrous magnesium sulfate ($MgSO_4$). The concentrated crude product was purified by column chromatography (SiO_2; EtOAc:hexanes = 1:8) to obtain **1** [R_f = 0.23; colorless oil; 180 mg, 0.70 mmol (yield = 90%)].^{1}H NMR [400 MHz, CD_3OD, δ (ppm)]: 5.88 (m, 1H), 5.11–5.28 (m, 2H), 3.79–4.46 (m, 4H), 1.43–1.65 (m, 15H). HRMS (*m/z*): $[M + Na]^+$ Calcd. for $C_{13}H_{23}NO_4Na$: 280.1519, found: 280.1528.

2 [*tert*-Butyl (*S*)-4-((*S,E*)-1-hydroxyhexadec-2-en-1-yl)-2,2-dimethyloxazolidine-3-carboxylate]. **2** was prepared following previously reported procedures with minor modifications.[2] A mixture of 1-pentadecene (75 μL, 0.28 mmol) and **1** (36 mg, 0.14 mmol) was stirred in anhydrous CH_2Cl_2 (2.5 mL). Second-generation Grubbs catalyst (2.4 mg, 3.0 μmol) was then added to the reaction mixture. After stirring at room temperature for 16 h, the reaction mixture was concentrated under the vacuum. The crude product was purified by column chromatography (SiO_2; from 0 to 10% EtOAc in hexanes) to obtain **2** [R_f = 0.30; yellow oil; 50 mg, 0.11 mmol (yield = 82%)]. ^{1}H NMR [400 MHz, CD_3OD, δ (ppm)]: 5.61–5.69 (m, 1H), 5.46–5.52 (m, 1H), 4.11 (t, J = 6.0 Hz, 1H), 3.91–4.04 (m, 3H), 3.79 (t, J = 4.0 Hz, 1H), 2.05 (m, 2 H), 1.29–1.54 (m, 37 H), 0.90 (t, J = 8.0 Hz, 3H). HRMS (*m/z*): $[M + Na]^+$ Calcd. for $C_{26}H_{49}NO_4Na$: 462.3554, found: 462.3552.

3 [*tert*-Butyl (4*S*)-4-((*E*)-1-acetoxyhexadec-2-en-1-yl)-2,2-dimethyloxazolidine-3-carboxylate]. **3** was produced with slight modifications of the previously reported procedures.[3] 4-Dimethylaminopyridine (2.8 mg, 23 μmol) was added to a solution of **2** (100 mg, 0.23 mmol) in a mixture of pyridine and CH_2Cl_2 (2:1, 3.0 mL). Acetyl chloride (25 μL, 0.35 mmol) was introduced dropwise to the reaction mixture. The mixture was stirred overnight at room temperature. After quenching the reaction by addition of CH_3OH (6.0 mL), the mixture was extracted with EtOAc (3x).

The combined organic phase was washed with brine (1x) and dried over anhydrous $MgSO_4$. The concentrated crude product was purified by column chromatography (SiO_2; EtOAc:hexanes = 1:9) to obtain **3** [R_f = 0.42; yellow oil; 92 mg, 0.19 mmol (yield = 52%)]. ^{1}H NMR [400 MHz, CD_3OD, δ (ppm)]: 5.68–5.79 (m, 1H), 5.54–5.65 (m, 1H), 5.39–5.50 (m, 1H), 3.87–4.13 (m, 3H), 2.01–2.06 (m, 5H), 1.44–1.54 (m, 15 H), 1.26–1.40 (m, 22 H), 0.90 (t, *J* = 6.0 Hz, 3H). HRMS (*m/z*): $[M + Na]^+$ Calcd. for $C_{28}H_{51}NO_5Na$: 504.3665, found: 504.3662.

3-*O*-Ac-SP [3-*O*-Acetylsphingosine, (2*S*,3*S*,*E*)-2-Amino-1-hydroxyoctadec-4-en-3-yl acetate]. 3-*O*-Ac-SP was synthesized according to previously reported procedures with modifications.[4] Trifluoroacetic acid (2.0 mL) was added into a solution of **3** (282 mg, 0.59 mmol) in a mixture of CH_2Cl_2 and ddH_2O (2:1, 3.0 mL) and then stirred overnight at room temperature. After quenching the reaction by addition of NaOH (1.0 M, aq), the mixture was extracted with CH_2Cl_2 (5x). The organic layer was collected, washed with brine (1x), and dried over anhydrous $MgSO_4$. The solution was concentrated under the vacuum and purified by column chromatography (SiO_2; from 0 to 20% acetone in EtOAc). The final product, **3-*O*-Ac-SP**, was precipitated out in hexanes [R_f = 0.19; white solid; 73 mg, 0.21 mmol (yield = 37%)]. ^{1}H NMR [400 MHz, CD_2Cl_2, δ (ppm)]: 6.08–6.19 (m, 1H), 5.71–5.81 (m, 1H), 5.45–5.55 (m, 1H), 4.25–4.34 (m, 1H), 3.65–3.88 (m, 3H), 2.62 (br, 2H), 2.01–2.09 (m, 2H), 1.98–2.00 (m, 3H), 1.27–1.39 (m, 22H), 0.88 (t, *J* = 8.0 Hz, 3H). ^{13}C NMR [100 MHz, CD_2Cl_2, δ (ppm)]: 171.1, 134.4, 129.4, 74.7, 62.8, 55.5, 32.7, 32.3, 30.1, 30.1, 30.1, 30.1, 30.0, 29.9, 29.8, 29.6, 29.6, 23.5, 23.1, 14.3. HRMS (*m/z*): $[M + Na]^+$ Calcd. for $C_{20}H_{39}NO_3Na$: 364.2822, found: 364.2827.

CACs. Compounds were dissolved in a buffered solution (20 mM HEPES, pH 7.4 or 20 mM HEPES, pH 7.4 or pH 6.8, 150 mM NaCl) with 1% v/v DMSO. Their CACs were measured by dynamic light scattering (DLS) at room temperature. A laser operating at 633 nm was used as a light source. The scattered intensity was detected at an angle of 173° to the incident beam. Averaged values of hydrodynamic radius (R_h) were calculated by a cumulant method using the software provided by the manufacturer (Zetasizer Software v7.12; Malvern Panalytical Ltd.). The CACs were obtained from a plot of the R_h values as a function of the concentrations of compounds in a logarithmic scale.

ITC. Solutions of **SP** or acetylsphingosines [200 μM; 20 mM HEPES, pH 7.4 (1% v/v DMSO)] and $A\beta_{40}$ {500 μM; 20 mM HEPES, pH 7.4 [1% w/w ammonium hydroxide (NH_4OH)]} were subjected to centrifugation for 5 min at 10,000 *g* prior to loading into the instrument. The solution of $A\beta_{40}$ in

the syringe was titrated into the solution containing compounds in the cell at 10 °C with 19 injections at a constant interval of 150 s (0.4 μL for the first injection and 2 μL for the following injections). The cell was continuously stirred at 750 rpm. The initial delay was 60 s and the reference power was 10 μcal/s. Dilution heat of $A\beta_{40}$ was also measured under the same experimental conditions above. The ITC thermogram and binding isotherm were shown after the subtraction of the dilution heat. The binding isotherm after baseline correction was fitted to the one-site binding model using the MicroCal PEAQ-ITC analysis software (Malvern Panalytical Ltd.).

2D ^{1}H–^{15}N SOFAST-HMQC NMR Experiments. Uniformly ^{15}N-labeled $A\beta_{40}$ was dissolved in NH_4OH (1% w/w, aq) and the resulting solution was lyophilized for 48 h and stored at –80 °C. To remove possible preformed aggregates, the lyophilized peptide was then redissolved in diluted NaOH (pH 10) and passed through a filter (0.22 μm). The solution of ^{15}N-labeled $A\beta_{40}$ (40 μM) was prepared with and without Zn(II) (40 μM) and compounds (2 μM or 100 μM; 1% v/v DMSO) in 20 mM HEPES, pH 7.4 (10% v/v D_2O). All spectra were acquired with 180 scans at 10 °C. Dimensions of the spectra were 1,024 (^{1}H) × 128 (^{15}N) points. Experimental data were processed with NMRpipe[5] and Sparky 3.115.[6] The ^{1}H and ^{15}N assignments of ^{15}N-labeled $A\beta_{40}$ were determined based on the previous results.[7,8] The chemical shift perturbation was calculated following the equation (eq 1).[9] Note that $\Delta\delta_H$ and $\Delta\delta_N$ represent the alterations in the chemical shifts of ^{1}H and ^{15}N of ^{15}N-labeled $A\beta_{40}$, respectively.

$$\Delta\delta_{NH} = \sqrt{(\Delta\delta_H)^2 + \left(\frac{\Delta\delta_N}{6.5}\right)^2} \qquad \text{(eq 1)}$$

Docking Studies. Flexible ligand docking studies using AutoDock Vina[10] were conducted against the $A\beta_{40}$ monomer [Protein Data Bank (PDB) ID: 2LFM] that was previously determined by NMR in an aqueous solution.[11] Two conformations of $A\beta_{40}$ (2 and 18) were selected from 20 conformations within the PDB file. The MM2 energy minimization in ChemBio3D 16.0 was used to optimize the structures of compounds prior to docking studies. The structures of $A\beta_{40}$ with compounds were prepared using AutoDock Tools and imported into PyRx,[12] which was used to run AutoDock Vina.[10] The exhaustiveness for the docking runs was set at 1,024. Docked models of compounds with $A\beta_{40}$ were visualized using Pymol 2.0.7.

Zn(II)-binding Studies. The solution of 2-aminoethanol (100 μM) was prepared in a mixture of ddH$_2$O and D$_2$O (9:1). Various concentrations of Zn(NO$_3$)$_2$·nH$_2$O (0.005, 0.01, 0.05, 0.10, 0.15, 0.32, 0.82, and 1.81 mM) were titrated into the solution of the ligand. Solvent suppression with the excitation sculpting and flip-back was performed to acquire the NMR spectra of the ligand in the presence of 3-(trimethylsilyl)propane-1-sulfonate sodium salt (DSS, 10 μM; Sigma-Aldrich) used as an internal reference. Changes in the ^{1}H chemical shift upon increasing the concentration of Zn(II) were plotted and fitted using the equation (eq 2) to obtain the K_d value of the ligand for Zn(II).[13] Note that $\Delta\delta$ and $\Delta\delta_\infty$ represent the change in the chemical shift and its maximum value, respectively. All measurements were conducted in triplicate.

$$\frac{\Delta\delta}{\Delta\delta_\infty} = \frac{K_d + [\text{Ligand}]_{total} + [\text{Zn(II)}]_{total} - \sqrt{(K_d + [\text{Ligand}]_{total} + [\text{Zn(II)}]_{total})^2 - 4 \times [\text{Ligand}]_{total} \times [\text{Zn(II)}]_{total}}}{2 \times [\text{Ligand}]_{total}} \quad \text{(eq 2)}$$

Solution Speciation Studies. The acidity constant (pK_a) for 2-aminoethanol was determined through UV–vis variable-pH titrations based on previously reported procedures.[14,15] The solution of a compound (400 μM; 10 mM NaOH, pH 12, 100 mM NaCl) was titrated with small aliquots of HCl to obtain at least 30 spectra in the pH range from 3 to 11. Utilizing the measured pK_a value, Cu(II)-binding properties of 2-aminoethanol were analyzed through UV–vis variable-pH titrations. Small aliquots of HCl were titrated into a solution containing the compound and CuCl$_2$ ([Cu(II)] = 200 μM; [2-aminoethanol] = 400 μM). At least 30 spectra were acquired in the range of pH 3–8. The values of pK_a and stability constants were calculated by the HypSpec program (Protonic Software, Leeds, UK).[16,17]

EPR Measurements. The solution of CuCl$_2$ (100 μM; 20 mM HEPES, pH 6.8, 150 mM NaCl) was prepared with 1% v/v DMSO, **SP**, and ***N*-Ac-SP** [400 μM (1% v/v DMSO)] in the absence and presence of Aβ_{16} (100 μM). The samples were incubated for 24 h at 37 °C with constant agitation and then transferred to EPR tubes. The EPR spectra of all frozen samples were recorded with the following experimental parameters: microwave frequency, 9.41 GHz; microwave power, 2 mW; modulation frequency, 100 kHz; modulation amplitude, 10 G; time constant, 0.01 ms; conversion time, 60 ms; sweep time, 120 s; number of scan, 4; temperature, 100 K. The acquired spectra were processed with baseline correction by subtracting a ninth-order polynomial. The *g*- and *A*-tensors were determined by numerical simulation of the experimental spectra using the EasySpin toolbox within MATLAB.[18] After spectral normalization by double integration following previously

reported procedures,[19–22] the simulated spectrum was obtained by weighted summation of the experimental spectra.

Aβ Aggregation Experiments. Aβ was dissolved in NH_4OH (1% w/w, aq) and the resulting solution was aliquoted, lyophilized overnight, and stored at –80 °C. A stock solution of Aβ was then prepared by dissolving the lyophilized peptide with NH_4OH (1% w/w, aq; 10 μL) and diluting with ddH_2O. All Aβ samples were prepared following previously reported procedures.[8,14] The concentration of the peptide solution was determined by measuring its absorbance at 280 nm (ε = 1,450 $M^{-1}cm^{-1}$ for $A\beta_{40}$; ε = 1,490 $M^{-1}cm^{-1}$ for $A\beta_{16}$ and $A\beta_{42}$). The buffered solution (20 mM HEPES, pH 6.8 or 7.4, 150 mM NaCl) was used for preparing Aβ samples. Compounds (final concentration, 10 μM or 100 μM; 1% v/v DMSO) were added to the samples of Aβ (final concentration, 25 μM) in the absence and presence of $CuCl_2$ or $ZnCl_2$ (final concentration, 25 μM) followed by incubation for 24 h at 37 °C with constant agitation.

Gel/Western Blot. Resultant Aβ species from the *in vitro* Aβ aggregation experiments were analyzed by gel/Western blot using an anti-Aβ antibody (6E10, Covance, Princeton, NJ, USA).[8,14] Each sample (10 μL) was separated on a 10–20% tricine gel (Invitrogen, Carlsbad, CA, USA). Following separation, the proteins were transferred onto nitrocellulose membranes and blocked with bovine serum albumin (BSA, 3% w/v; Sigma-Aldrich) in Tris-buffered saline (TBS) containing 0.1% v/v Tween-20 (Sigma-Aldrich) (TBS-T) for 4 h at room temperature or overnight at 4 °C. The membranes were incubated with 6E10 (1:2,000) in the solution of BSA (2% w/v in TBS-T) for 4 h at room temperature. After washing with TBS-T (3x, 10 min), a horseradish peroxidase-conjugated goat anti-mouse secondary antibody (1:5,000 in 2% w/v BSA in TBS-T; Cayman Chemical Company, Ann Arbor, MI, USA) was added for 2 h at room temperature. A homemade ECL kit[8,14,23] was used to visualize gel/Western blots on a ChemiDoc MP Imaging System (Bio-Rad).

TEM. Samples for TEM were prepared following previously reported methods.[14,24,25] Glow-discharged grids (Formvar/Carbon 300-mesh; Electron Microscopy Sciences, Hatfield, PA, USA) were treated with Aβ samples (25 μM; 7.5 μL) for 2 min at room temperature. Excess sample was removed using filter paper followed by washing with ddH_2O (3x). Each grid incubated with uranyl acetate (1% w/v ddH_2O; 7.5 μL) for 2 min was blotted off and dried overnight at room temperature. Locations of the samples on the grids were randomly picked for taking more than 20 images from

each grid.

Cell Viability Studies. SH-SY5Y cells were maintained in media containing 50% v/v minimum essential medium (MEM; GIBCO, Grand Island, NY, USA) and 50% v/v F12 (GIBCO) and supplemented with 10% v/v fetal bovine serum (Sigma-Aldrich) and 100 U/mL penicillin with 100 mg/mL streptomycin (GIBCO). The cells were grown and maintained at 37 °C in a humidified atmosphere with 5% CO_2. Cell viability was determined by the MTT assay. Cells were seeded in a 96-well plate (15,000 cells/100 μL) and treated with the samples {[compound] = 10 μM (0.2% v/v DMSO); [$CuCl_2$ or $ZnCl_2$] = 10 μM; [Aβ_{42}] = 10 μM}. After 24 h incubation, MTT [5 mg/mL in PBS (pH 7.4, GIBCO); 25 μL] was added to each well and the plate was incubated for 4 h at 37 °C. Formazan produced by cells was solubilized using an acidic solution of *N*,*N*-dimethylformamide (DMF; pH 4.5, 50% v/v, aq) and sodium dodecyl sulfate (SDS; 20% w/v) overnight at room temperature in the dark. Absorbance was measured at 600 nm by a microplate reader (Molecular Devices). Cell viability was calculated relative to that of the cells treated with an equivalent amount of the buffered solution with 0.2% v/v DMSO (20 mM HEPES, pH 7.4 or pH 6.8, 150 mM NaCl). IC_{50} values of compounds in a buffered solution (20 mM HEPES, pH 6.8 or 7.4, 150 mM NaCl) were calculated from the titration curves as a function of the concentrations of compounds (1–100 μM; 0.2% v/v DMSO) using Prism 7 (GraphPad). All measurements were conducted in triplicate.

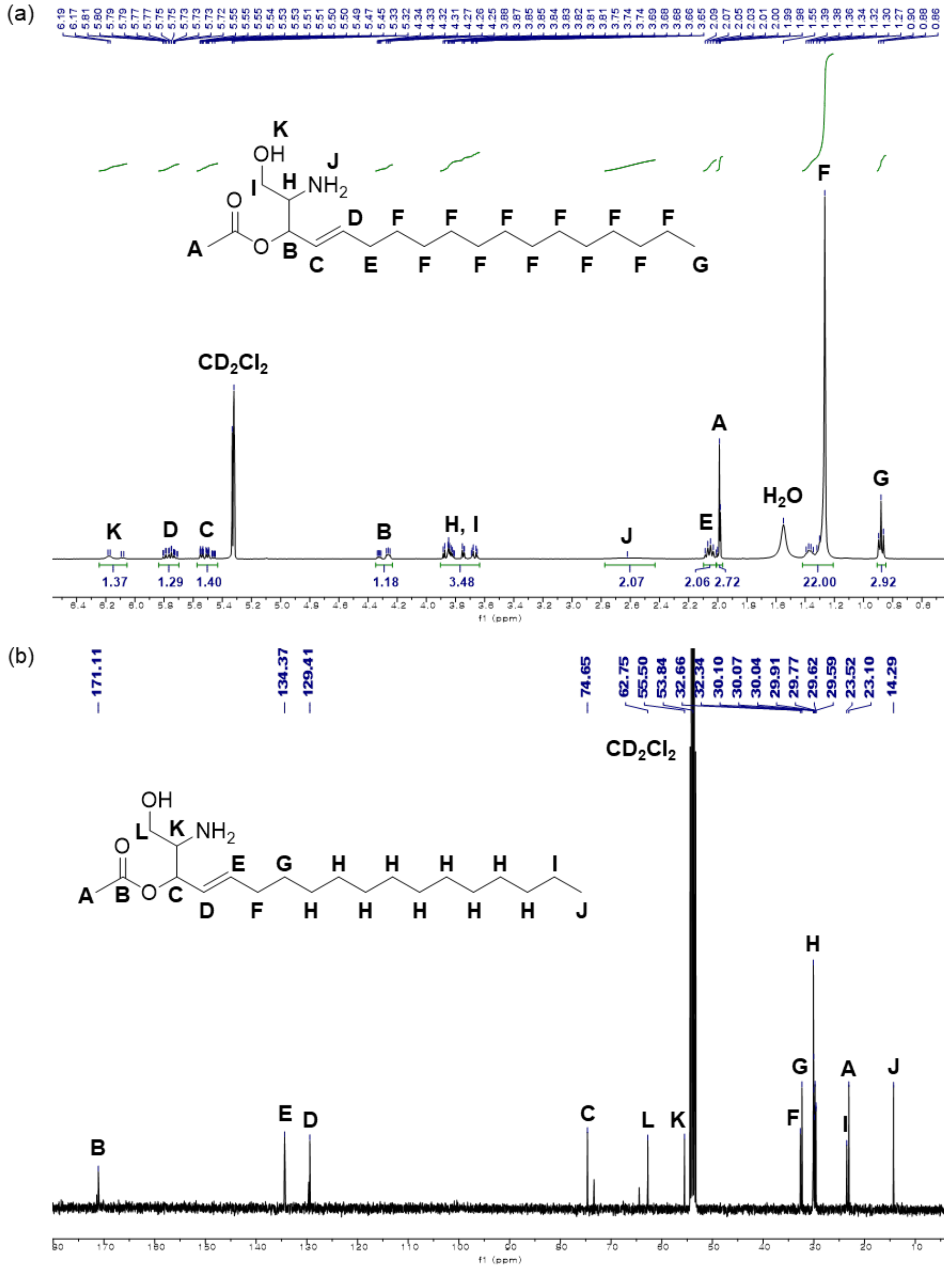

Fig. S1 NMR [(a) ^{1}H (400 MHz) and (b) ^{13}C (100 MHz)] spectra of **3-*O*-Ac-SP** (CD_2Cl_2).

(a)

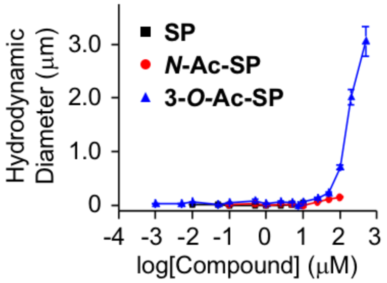

Compound	CAC (μM)
**SP**	4.3 ± 1.3
***N*-Ac-SP**	12.0 ± 4.9
**3-*O*-Ac-SP**	56.9 ± 10.9

(b)

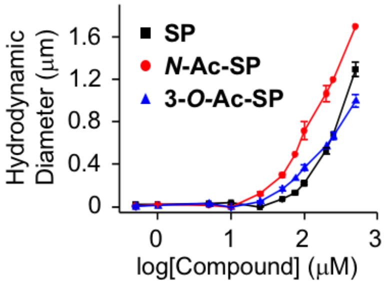

Compound	CAC (μM)
**SP**	79.6 ± 1.1
***N*-Ac-SP**	26.0 ± 0.3
**3-*O*-Ac-SP**	29.5 ± 0.7

(c)

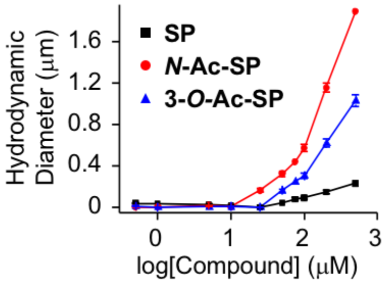

Compound	CAC (μM)
**SP**	26.4 ± 0.6
***N*-Ac-SP**	26.6 ± 0.4
**3-*O*-Ac-SP**	30.8 ± 0.2

Fig. S2 Aggregation of **SP** and the acetylsphingosines observed by DLS. Their mean hydrodynamic diameters (left) and CACs (right) were determined in three different buffered solutions: (a) 20 mM HEPES, pH 7.4; (b) 20 mM HEPES, pH 7.4, 150 mM NaCl; (c) 20 mM HEPES, pH 6.8, 150 mM NaCl.

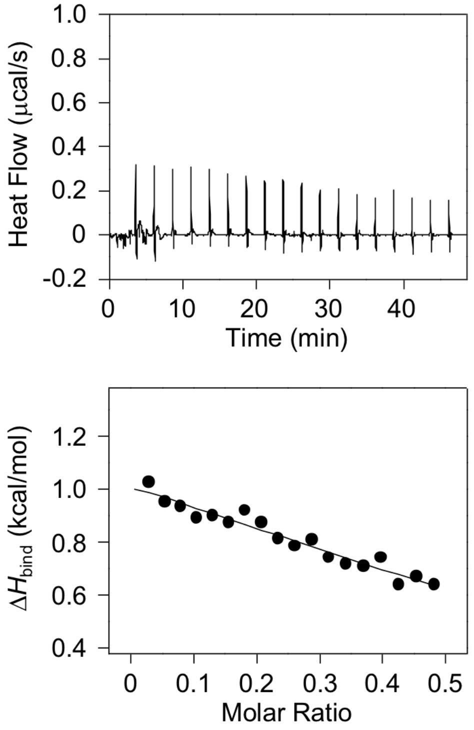

Fig. S3 ITC thermogram (top) and binding isotherm (bottom) of **SP** with Aβ_{40} monomer. The black line indicates a fit curve. Conditions: [Aβ_{40}] = 500 μM; [**SP**] = 200 μM (1% v/v DMSO); 20 mM HEPES, pH 7.4; 10 °C.

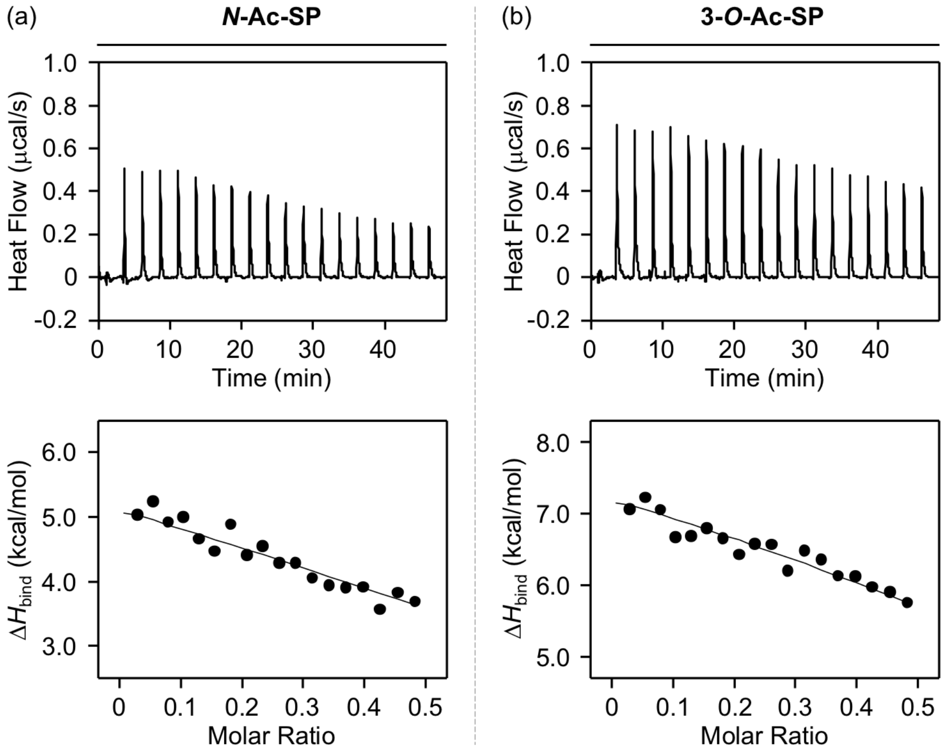

Fig. S4 ITC thermograms (top) and binding isotherms (bottom) of (a) ***N*-Ac-SP** and (b) **3-*O*-Ac-SP** with Aβ_{40} monomer. The black lines indicate fit curves. Conditions: [Aβ_{40}] = 500 μM; [acetylsphingosine] = 200 μM (1% v/v DMSO); 20 mM HEPES, pH 7.4; 10 °C.

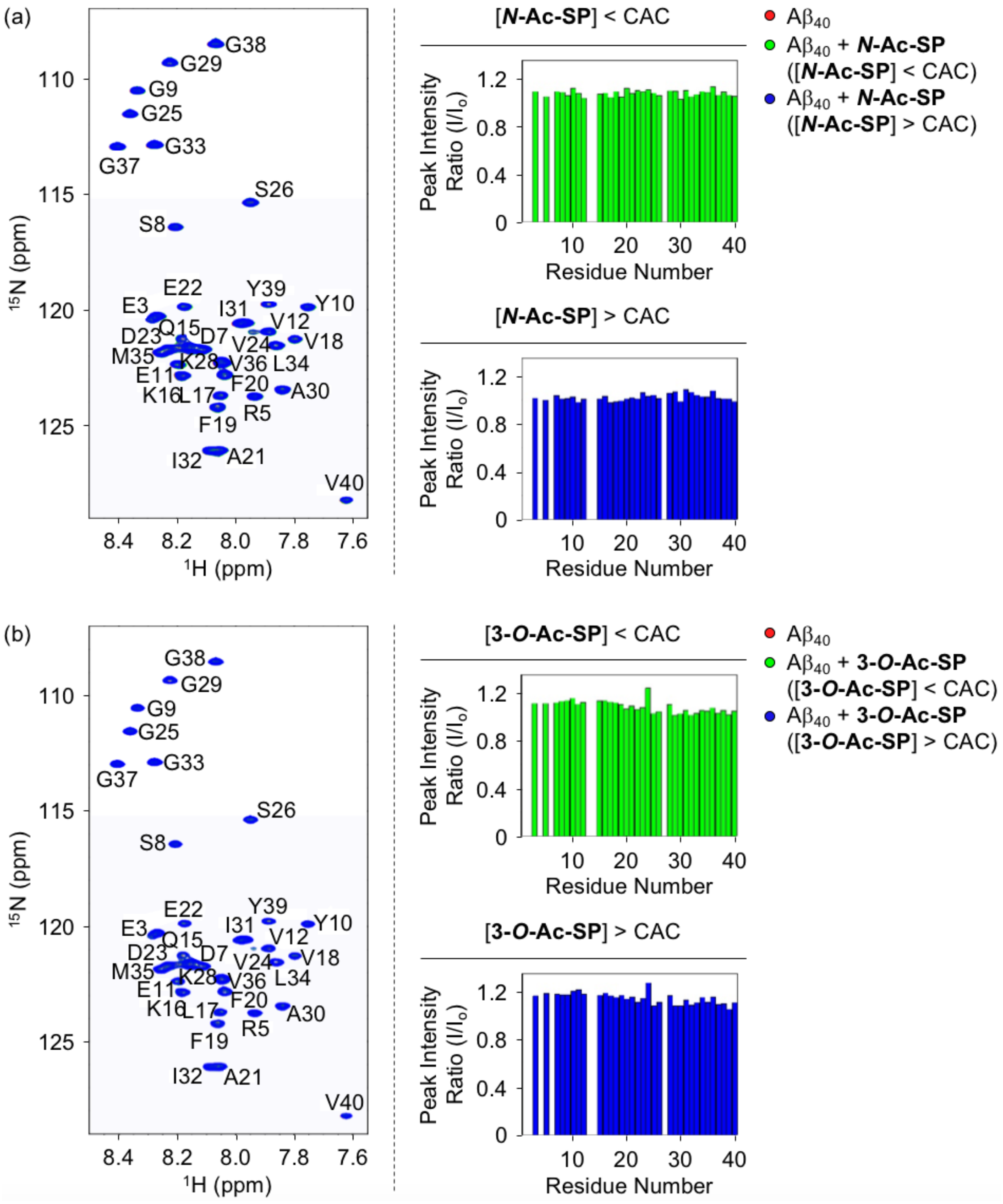

Fig. S5 2D ^{1}H–^{15}N SOFAST-HMQC NMR spectra and peak intensity ratios of ^{15}N-labeled Aβ_{40} upon treatment of (a) ***N*-Ac-SP** and (b) **3-*O*-Ac-SP** at the concentrations below or above their CACs. Conditions: [^{15}N-labeled Aβ_{40}] = 40 μM; [acetylsphingosine] = 2 μM (below the CAC) or 100 μM (above the CAC) (1% v/v DMSO); 20 mM HEPES, pH 7.4; 10% v/v D_2O; 10 °C.

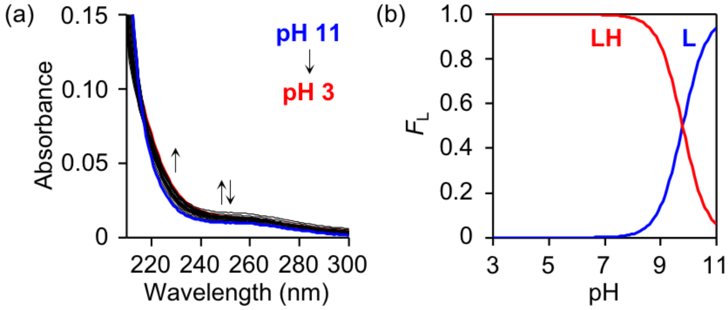

(c)

	pK_a
L + H ⇄ LH	9.79(0)

Fig. S6 Solution speciation studies of 2-aminoethanol (L). The UV–vis variable-pH titration spectra (a), the solution speciation diagram (b) (F_L = fraction of species at given pH), and the pK_a value (c) of the compound are depicted. The error in the last digit is shown in parentheses. Charges are omitted for clarity. Conditions: [L] = 400 μM; room temperature; I = 0.10 M NaCl.

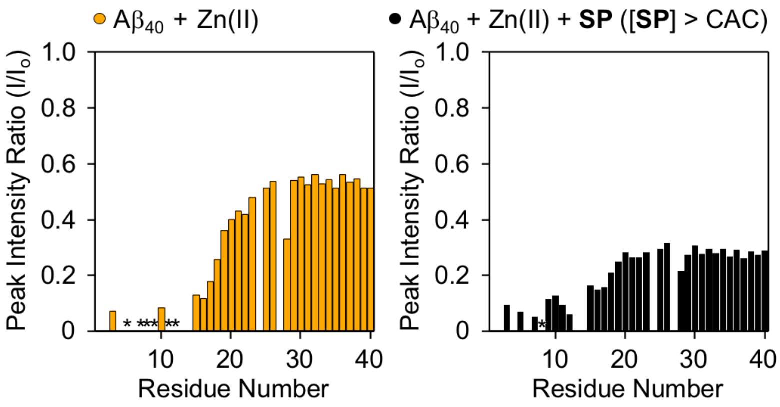

Fig. S7 Peak intensity ratios of ^{15}N-labeled Aβ_{40} with Zn(II) in the absence and presence of **SP** at the concentration above the CAC obtained from the 2D ^{1}H–^{15}N SOFAST-HMQC NMR spectra shown in Fig. 5a. Upon addition of Zn(II) into the solution of ^{15}N-labeled Aβ_{40} with and without **SP**, the NMR signals of the amino acid residues indicated in asterisks(*) disappeared. Conditions: [^{15}N-labeled Aβ_{40}] = 40 μM; [Zn(II)] = 40 μM; [**SP**] = 100 μM (above the CAC) (1% v/v DMSO); 20 mM HEPES, pH 7.4; 10% v/v D_2O; 10 °C.

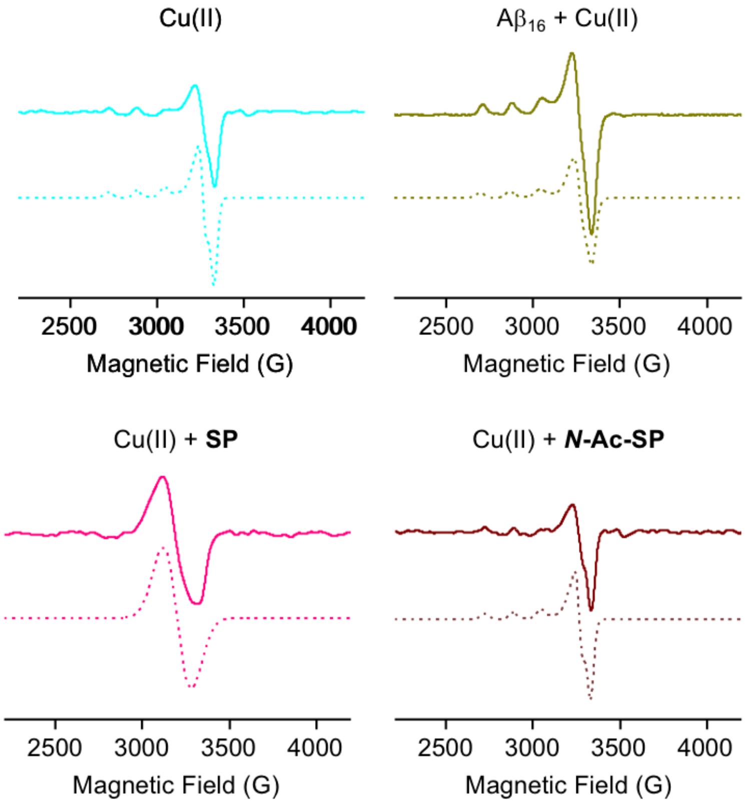

	$g_{\perp}$	$g_{\parallel}$	$A_{\perp}$	$A_{\parallel}$
Cu(II)	2.05	2.27	5.54	157.78
Cu(II) + Aβ_{16}	2.05	2.28	6.96	167.75
Cu(II) + **SP**	2.10	2.10	10.55	10.55
Cu(II) + ***N*-Ac-SP**	2.05	2.27	5.54	157.78

Fig. S8 Experimental (solid line) and simulated (dashed line) EPR spectra of the Cu(II) samples with and without Aβ_{16}, **SP**, and ***N*-Ac-SP**. Spin-Hamiltonian parameters, including *g*- and *A*-tensors, obtained by numerical simulation of the experimental EPR spectra were summarized in the table. Conditions: [Aβ_{16}] = 100 μM; [Cu(II)] = 100 μM; [compound] = 400 μM (1% v/v DMSO); 20 mM HEPES, pH 6.8, 150 mM NaCl; 37 °C; 24 h; constant agitation. The EPR spectra were recorded with the following experimental parameters: microwave frequency, 9.41 GHz; microwave power, 2 mW; modulation frequency, 100 kHz; modulation amplitude, 10 G; time constant, 0.01 ms; conversion time, 60 ms; sweep time, 120 s; number of scan, 4; temperature, 100 K.

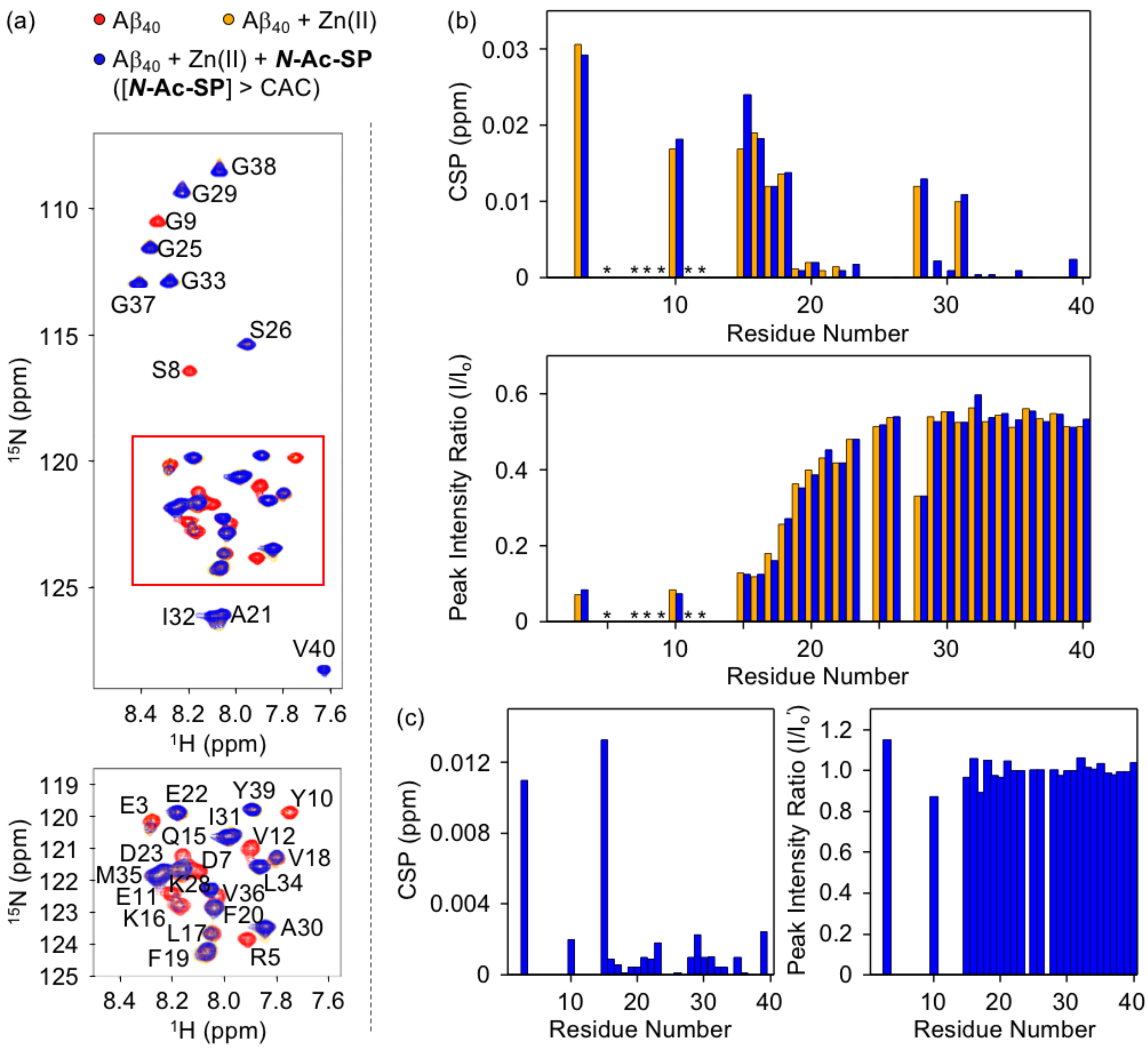

Fig. S9 2D ^{1}H–^{15}N SOFAST-HMQC NMR analysis of the samples containing ^{15}N-labeled $A\beta_{40}$, Zn(II), and ***N*-Ac-SP** at the concentration above the CAC. (a) 2D NMR spectra of ^{15}N-labeled $A\beta_{40}$ with and without Zn(II) and ***N*-Ac-SP** at the concentration above the CAC (top). Bottom: Expanded view of the region of the spectra highlighted in red. (b) CSPs and peak intensity ratio of Zn(II)-treated ^{15}N-labeled $A\beta_{40}$ with and without ***N*-Ac-SP** at the concentration above the CAC, relative to metal-free ^{15}N-labeled $A\beta_{40}$. Upon addition of Zn(II) into the solution of ^{15}N-labeled $A\beta_{40}$ with and without ***N*-Ac-SP**, the NMR signals of the amino acid residues indicated in asterisks(*) disappeared. (c) CSPs and peak intensity ratio of Zn(II)-added ^{15}N-labeled $A\beta_{40}$ in the presence of ***N*-Ac-SP** at the concentration above the CAC, compared to Zn(II)-treated ^{15}N-labeled $A\beta_{40}$. Conditions: [^{15}N-labeled $A\beta_{40}$] = 40 μM; [Zn(II)] = 40 μM; [***N*-Ac-SP**] = 100 μM (above the CAC) (1% v/v DMSO); 20 mM HEPES, pH 7.4; 10% v/v D_2O; 10 °C.

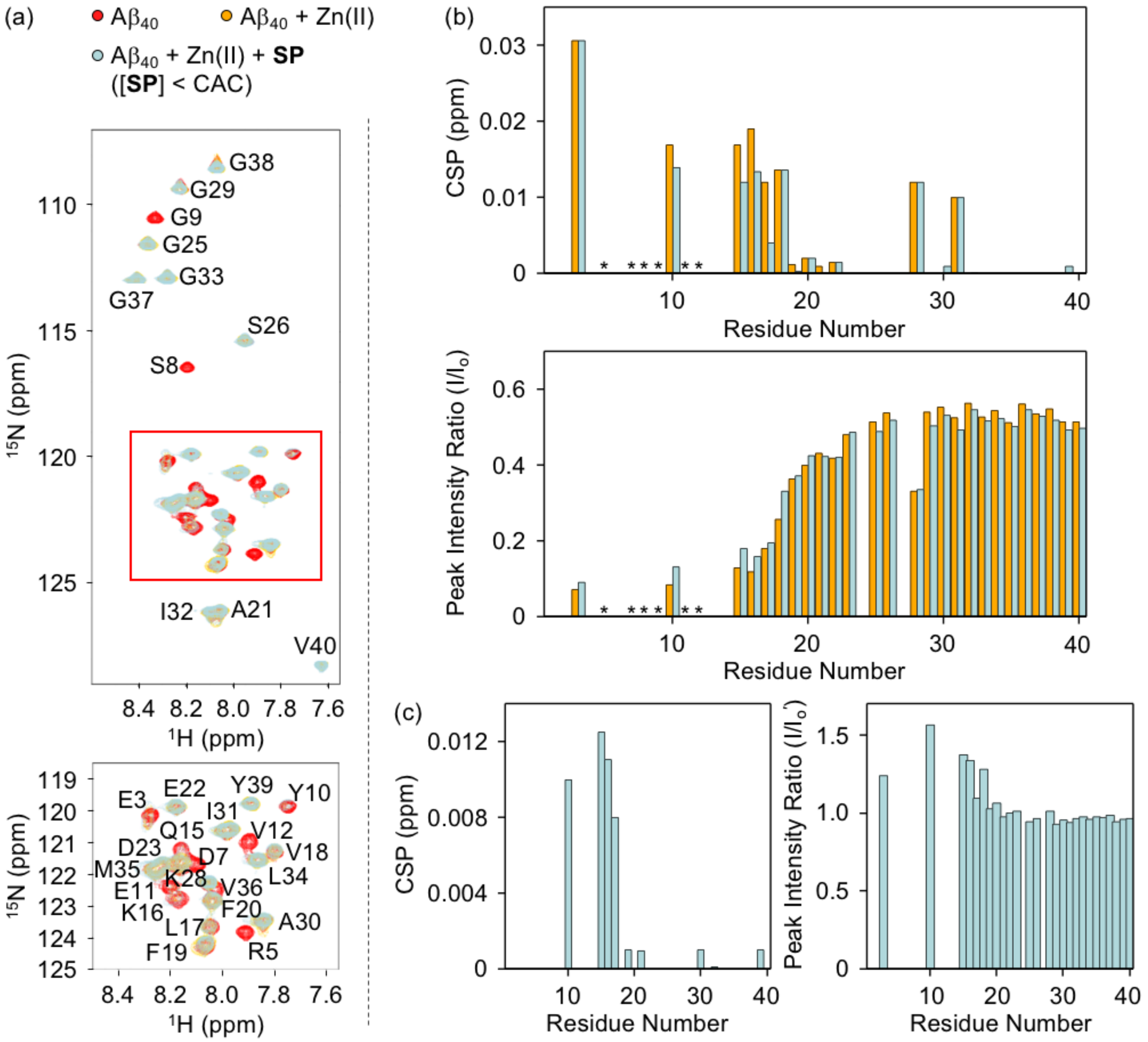

Fig. S10 2D ^{1}H–^{15}N SOFAST-HMQC NMR analysis of the samples containing ^{15}N-labeled Aβ_{40}, Zn(II), and **SP** at the concentration below the CAC. (a) 2D NMR spectra of ^{15}N-labeled Aβ_{40} with and without Zn(II) and **SP** at the concentration below the CAC (top). Bottom: Expanded view of the region of the spectra highlighted in red. (b) CSPs and peak intensity ratio of Zn(II)-treated ^{15}N-labeled Aβ_{40} with and without **SP** at the concentration below the CAC, relative to metal-free ^{15}N-labeled Aβ_{40}. Upon addition of Zn(II) into the solution of ^{15}N-labeled Aβ_{40} with and without **SP**, the NMR signals of the amino acid residues indicated in asterisks(*) disappeared. (c) CSPs and peak intensity ratio of Zn(II)-added ^{15}N-labeled Aβ_{40} in the presence of **SP** at the concentration below the CAC, compared to Zn(II)-treated ^{15}N-labeled Aβ_{40}. Conditions: [^{15}N-labeled Aβ_{40}] = 40 μM; [Zn(II)] = 40 μM; [**SP**] = 2 μM (below the CAC) (1% v/v DMSO); 20 mM HEPES, pH 7.4; 10% v/v D_2O; 10 °C.

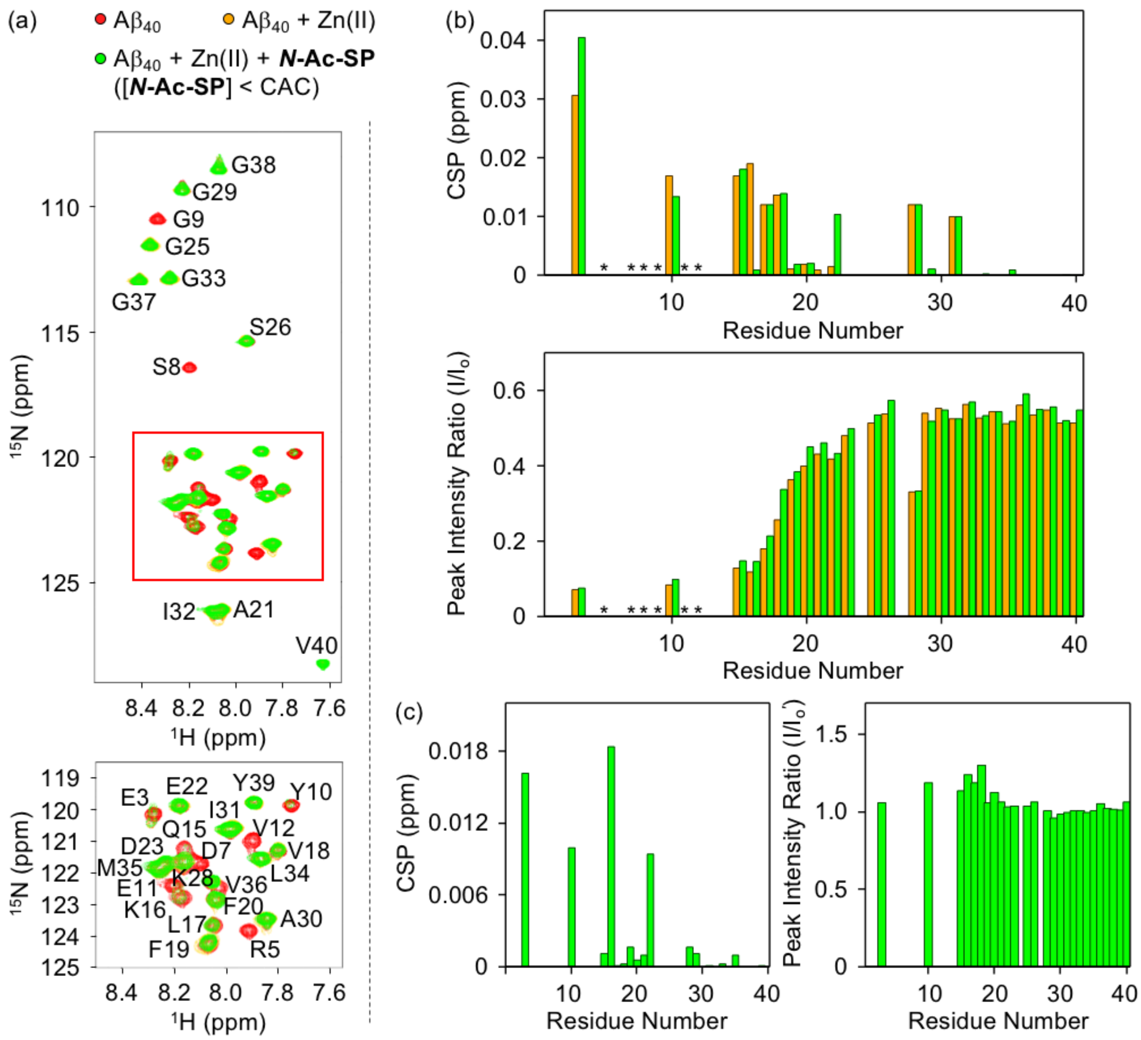

Fig. S11 2D ^{1}H–^{15}N SOFAST-HMQC NMR studies of the samples containing ^{15}N-labeled Aβ_{40}, Zn(II), and ***N*-Ac-SP** at the concentration below the CAC. (a) 2D NMR spectra of ^{15}N-labeled Aβ_{40} with and without Zn(II) and ***N*-Ac-SP** at the concentration below the CAC (top). Bottom: Expanded view of the region of the spectra highlighted in red. (b) CSPs and peak intensity ratio of Zn(II)-treated ^{15}N-labeled Aβ_{40} with and without ***N*-Ac-SP** at the concentration below the CAC, relative to metal-free ^{15}N-labeled Aβ_{40}. Upon addition of Zn(II) into the solution of ^{15}N-labeled Aβ_{40} with and without ***N*-Ac-SP**, the NMR signals of the amino acid residues indicated in asterisks(*) disappeared. (c) CSPs and peak intensity ratio of Zn(II)-added ^{15}N-labeled Aβ_{40} in the presence of ***N*-Ac-SP** at the concentration below the CAC, compared to Zn(II)-treated ^{15}N-labeled Aβ_{40}. Conditions: [^{15}N-labeled Aβ_{40}] = 40 μM; [Zn(II)] = 40 μM; [***N*-Ac-SP**] = 2 μM (below the CAC) (1% v/v DMSO); 20 mM HEPES, pH 7.4; 10% v/v D_2O; 10 °C.

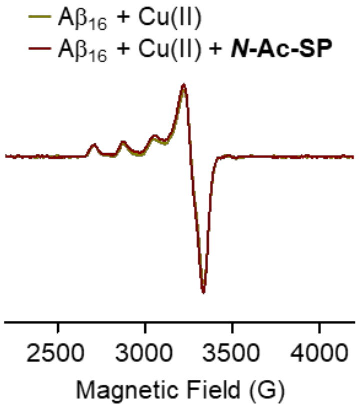

Fig. S12 EPR spectra of the samples containing Cu(II) and $A\beta_{16}$ in the absence and presence of ***N*-Ac-SP**. Conditions: [$A\beta_{16}$] = 100 μM; [Cu(II)] = 100 μM; [***N*-Ac-SP**] = 400 μM (above the CAC) (1% v/v DMSO); 20 mM HEPES, pH 6.8, 150 mM NaCl; 37 °C; 24 h; constant agitation. The EPR spectra were recorded with the following experimental parameters: microwave frequency, 9.41 GHz; microwave power, 2 mW; modulation frequency, 100 kHz; modulation amplitude, 10 G; time constant, 0.01 ms; conversion time, 60 ms; sweep time, 120 s; number of scan, 4; temperature, 100 K.

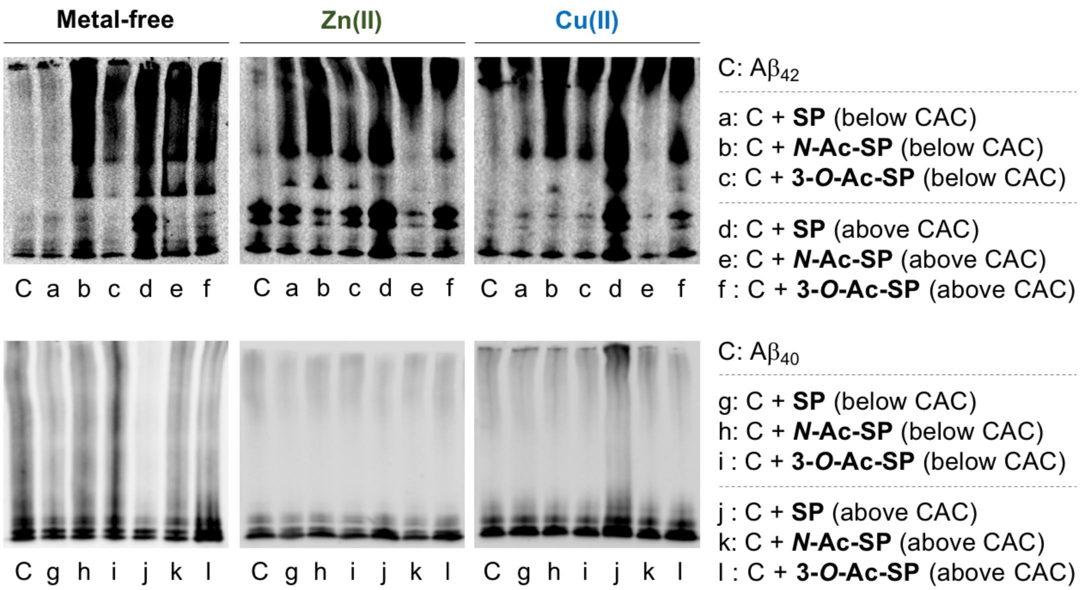

Fig. S13 Original gel images from Fig. 6, 7, S14, and S15.

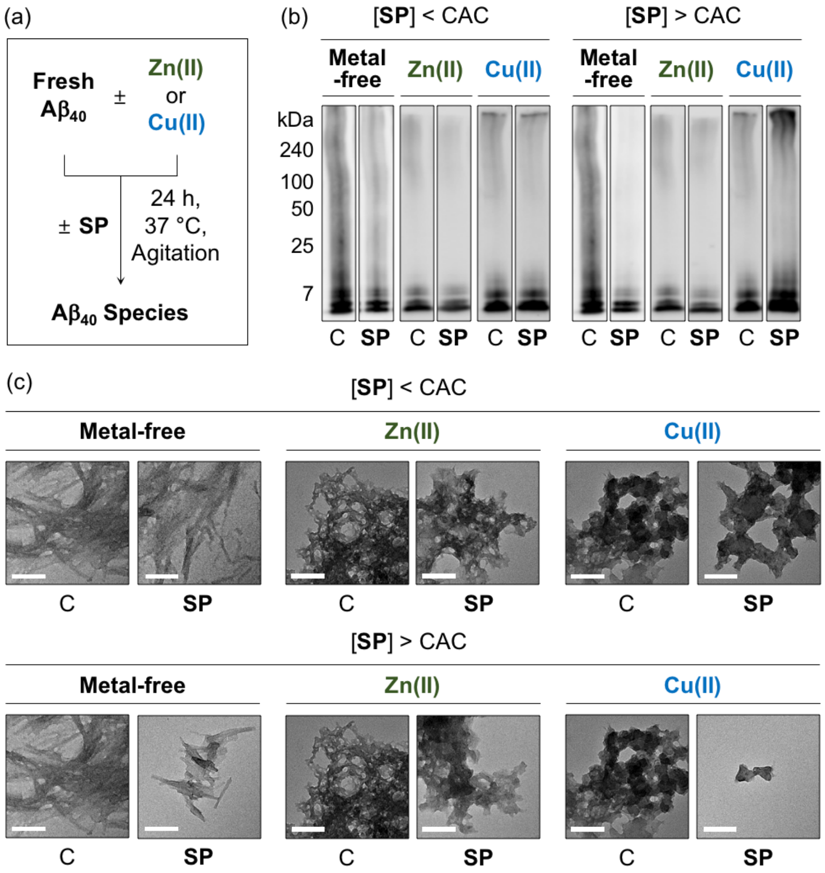

Fig. S14 Impact of **SP** on metal-free and metal-induced Aβ_{40} aggregation. (a) Scheme of Aβ_{40} aggregation experiments. (b) Analysis of the size distribution of the resultant Aβ_{40} species by gel/Western blot using an anti-Aβ antibody (6E10). Lanes: (C) Aβ_{40} ± Zn(II) or Cu(II); (**SP**) C + **SP**. The original gel images are shown in Fig. S13. (c) Morphologies of the resultant Aβ_{40} aggregates from (b) detected by TEM. Conditions: [Aβ_{40}] = 25 μM; [Zn(II) or Cu(II)] = 25 μM; [compound] = 10 μM (below the CAC) or 100 μM (above the CAC) (1% v/v DMSO); 20 mM HEPES, pH 7.4 [for metal-free and Zn(II)-containing samples] or pH 6.8 [for Cu(II)-containing samples], 150 mM NaCl; 37 °C; 24 h; constant agitation. Scale bars = 200 nm.

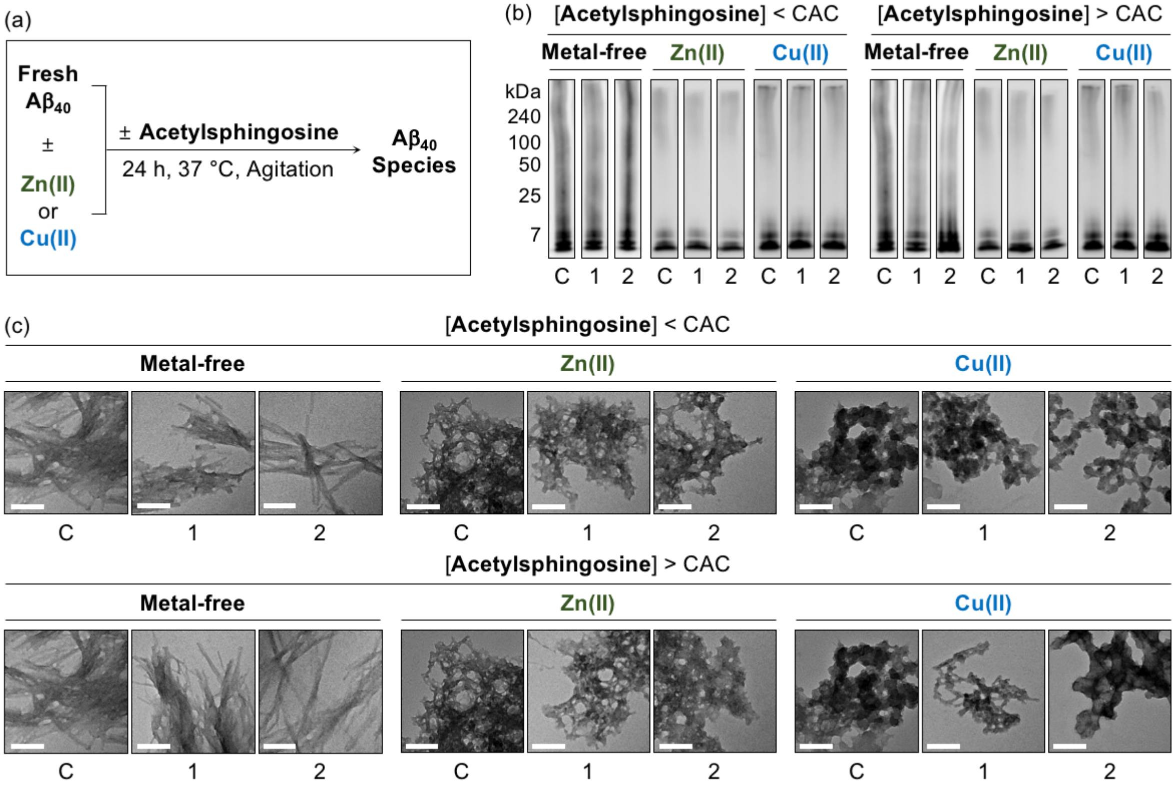

Fig. S15 Influence of ***N*-Ac-SP** and **3-*O*-Ac-SP** on the aggregation of metal-free Aβ_{40} and metal–Aβ_{40}. (a) Scheme of Aβ_{40} aggregation experiments. (b) Analysis of the size distribution of the resultant Aβ_{40} species by gel/Western blot using an anti-Aβ antibody (6E10). Lanes: (C) Aβ_{40} ± Zn(II) or Cu(II); (1) C + ***N*-Ac-SP**; (2) C + **3-*O*-Ac-SP**. The original gel images are shown in Fig. S13. (c) Morphologies of the resultant Aβ_{40} aggregates from (b) monitored by TEM. Conditions: [Aβ_{40}] = 25 μM; [Zn(II) or Cu(II)] = 25 μM; [acetylsphingosine] = 10 μM (below the CAC) or 100 μM (above the CAC) (1% v/v DMSO); 20 mM HEPES, pH 7.4 [for metal-free and Zn(II)-containing samples] or pH 6.8 [for Cu(II)-containing samples], 150 mM NaCl; 37 °C; 24 h; constant agitation. Scale bars = 200 nm.

(a)

pH	IC_{50} (μM) SP	*N*-Ac-SP	3-*O*-Ac-SP
6.8	21.8 ± 0.4	47.4 ± 1.0	n.d.[a]
7.4	20.9 ± 0.5	39.8 ± 0.7	n.d.[a]

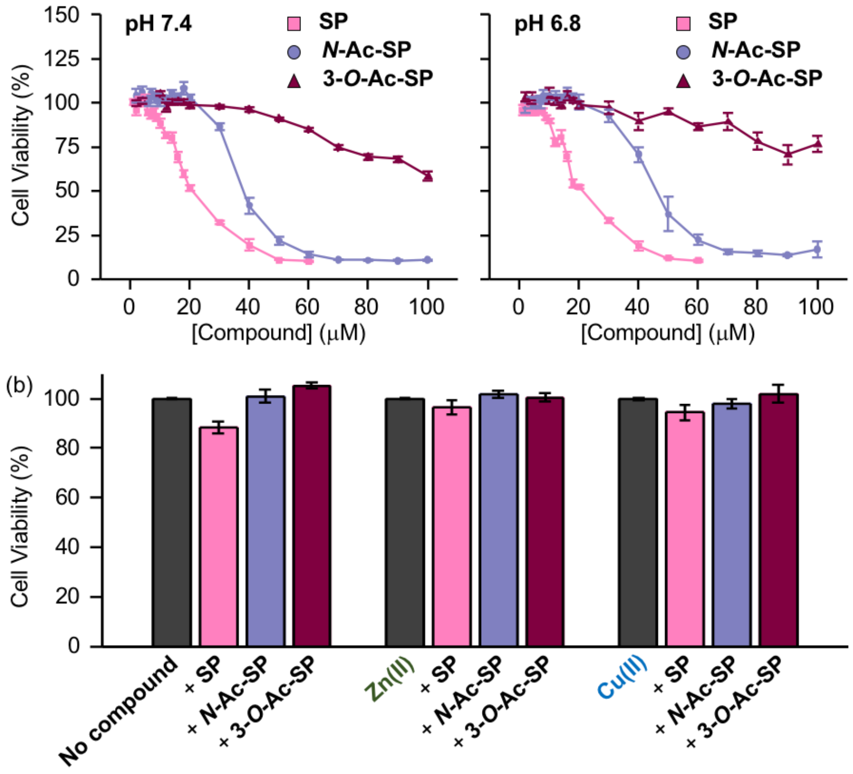

Fig. S16 Toxicity of **SP** and the acetylsphingosines in the absence and presence of metal ions in SH-SY5Y cells. Cell viability, determined by the MTT assay, was calculated in comparison to that of the cells treated with an equivalent amount of the buffered solution (20 mM HEPES, pH 7.4 or pH 6.8, 150 mM NaCl) with 0.2% v/v DMSO. Error bars indicate the standard error from three independent experiments. (a) IC_{50} values of the compounds that were calculated from cell survival upon treatment of **SP** and the acetylsphingosines in two different buffered solutions. [a]n.d., not determined. Cells incubated with **3-*O*-Ac-SP** indicated the viability greater than 55% up to 100 μM. Conditions: [compound] = 1–100 μM (0.2% v/v DMSO); 20 mM HEPES, pH 7.4 or pH 6.8, 150 mM NaCl. (b) Survival (%) of the cells incubated with **SP** or the acetylsphingosines with and without Zn(II) or Cu(II). Conditions: [Zn(II) or Cu(II)] = 10 μM; [compound] = 10 μM (0.2% v/v DMSO); 20 mM HEPES, pH 7.4 [for metal-untreated and Zn(II)-containing samples] or pH 6.8 [for Cu(II)-containing samples], 150 mM NaCl.

References

1. I. Ojima and E. S. Vidal, *J. Org. Chem.*, 1998, **63**, 7999.
2. P. Sanllehí, J.-L. Abad, J. Bujons, J. Casas and A. Delgado, *Eur. J. Med. Chem.*, 2016, **123**, 905.
3. P. Wisse, H. Gold, M. Mirzaian, M. J. Ferraz, G. Lutteke, R. J. B. H. N. van den Berg, H. van den Elst, J. Lugtenburg, G. A. van der Marel, J. M. F. G. Aerts, J. D. C. Codée and H. S. Overkleeft, *Eur. J. Org. Chem.*, 2015, 2661.
4. M. Taguchi, K. Sugimoto, K.-i. Goda, T. Akama, K. Yamamoto, T. Suzuki, Y. Tomishima, M. Nishiguchi, K. Arai, K. Takahashi and T. Kobori, *Bioorg. Med. Chem. Lett.*, 2003, **13**, 1963.
5. F. Delaglio, S. Grezesiek, G. W. Vuister, G. Zhu, J. Pfeifer and A. Bax, *J. Biomol. NMR*, 1995, **6**, 277.
6. Sparky 3, https://www.cgl.ucsf.edu/home/sparky/, (accessed December 2020).
7. K. J. Korshavn, M. Jang, Y. J. Kwak, A. Kochi, S. Vertuani, A. Bhunia, S. Manfredini, A. Ramamoorthy and M. H. Lim, *Sci. Rep.*, 2015, **5**.
8. M. W. Beck, J. S. Derrick, J.-M. Suh, M. Kim, K. J. Korshavn, R. A. Kerr, W. J. Cho, S. D. Larsen, B. T. Ruotolo, A. Ramamoorthy and M. H. Lim, *ChemMedChem*, 2017, **12**, 1828.
9. F. A. A. Mulder, D. Schipper, R. Bott and R. Boelens, *J. Mol. Biol.*, 1999, **292**, 111.
10. O. Trott and A. J. Olson, *J. Comput. Chem.*, 2010, **31**, 455.
11. S. Vivekanandan, J. R. Brender, S. Y. Lee and A. Ramamoorthy, *Biochem. Biophys. Res. Commun.*, 2011, **411**, 312.
12. L. K. Wolf, *Chem. Eng. News*, 2009, **87**, 31.
13. P. Thordarson, *Chem. Soc. Rev.*, 2011, **40**, 1305.
14. Y. Yi, J. Han, M. H. Park, N. Park, E. Nam, H. K. Jin, J.-s. Bae and M. H. Lim, *Chem. Commun.*, 2019, **55**, 5847.
15. J. S. Derrick, R. A. Kerr, K. J. Korshavn, M. J. McLane, J. Kang, E. Nam, A. Ramamoorthy, B. T. Ruotolo and M. H. Lim, *Inorg. Chem.*, 2016, **55**, 5000.
16. L. Alderighi, P. Gans, A. Lenco, D. Peters, A. Sabatini and A. Vacca, *Coord. Chem. Rev.*, 1999, **184**, 311.
17. P. Gans, A. Sabatini and A. Vacca, *Anal. Chim.*, 1999, **89**, 45.
18. S. Stoll and A. Schweiger, *J. Magn. Reson.*, 2006, **178**, 42.
19. X. Zhang, P. Cekan, S. T. Sigurdsson and P. Z. Qin, *Meth. Enzymol.*, 2009, **469**, 303.
20. S. C. Drew, *Appl. Magn. Reson.*, 2015, **46**, 1041.
21. S. C. Drew, S. L. Leong, C. L. Pham, D. J. Tew, C. L. Masters, L. A. Miles, R. Cappai and K. J. Barnham, *J. Am. Chem. Soc.*, 2008, **130**, 7766.

22. G. J. Troup, L. Navarini, F. S. Liverani and S. C. Drew, *PLoS One*, 2015, **10**, e0122834.
23. D. D. Mruk and C. Y. Cheng, *Spermatogenesis*, 2011, **1**, 121.
24. E. H. Nielsen, M. Nybo and S.-E. Svehag, *Meth. Enzymol.*, 1999, **309**, 491.
25. C. G. Evans, S. Wisén and J. E. Gestwicki, *J. Biol. Chem.*, 2006, **281**, 33182.
